# Correction: GLI1 facilitates collagen-induced arthritis in mice by collaborative regulation of DNA methyltransferases

**DOI:** 10.7554/eLife.98284

**Published:** 2024-03-28

**Authors:** Gaoran Ge, Qianping Guo, Ying Zhou, Wenming Li, Wei Zhang, Jiaxiang Bai, Qing Wang, Huaqiang Tao, Wei Wang, Zhen Wang, Minfeng Gan, Yaozeng Xu, Huilin Yang, Bin Li, Dechun Geng

**Keywords:** Mouse

Ge G, Guo Q, Zhou Y, Li W, Zhang W, Bai J, Wang Q, Tao H, Wang W, Wang Z, Gan M, Xu Y, Yang H, Li B, Geng D. 2023. GLI1 facilitates collagen-induced arthritis in mice by collaborative regulation of DNA methyltransferases. *eLife* **12**:e92142. doi: 10.7554/eLife.92142.

Published 6 November 2023

Whilst organising the data from this study we discovered errors in the sizing of the images shown in Figure 3N. We also could not locate some of the original raw images supporting the published images from Figure 3D and Figure 3N. We are therefore correcting Figure 3D (RANKL panel) and Figure 3N (all panels) with images from replicate experiments. These changes do not affect the conclusions of the article.

We have also updated the published paper to now include the raw microscopy images from Figure 3 as Figure 3—source data 2. We have updated the figure legend for Figure 3, to include the following reference to this source data:

**Source data 2**. Raw microscopy images for Figure 3.

The corrected Figure 3 is shown here:

**Figure 1:**
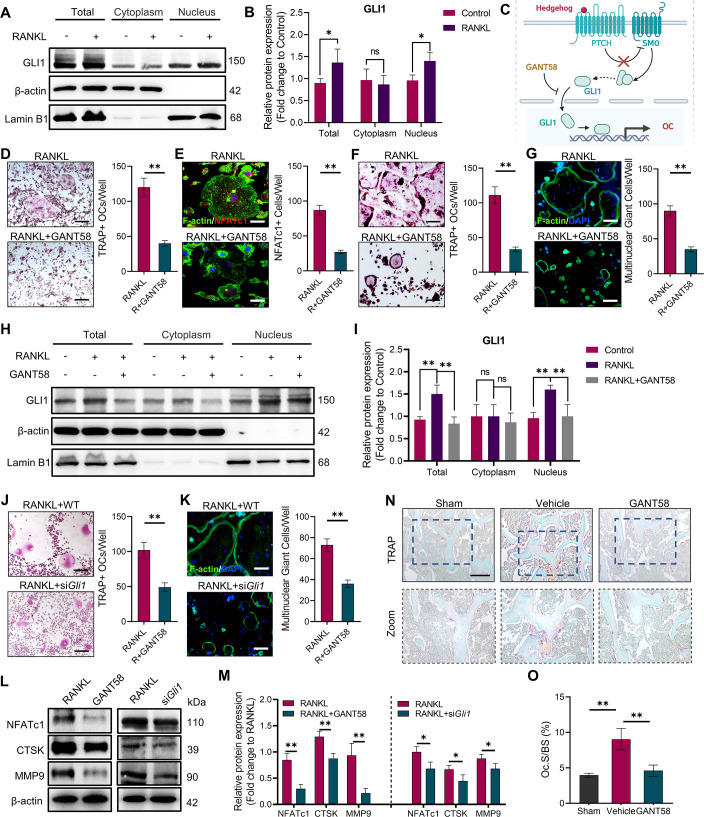


The originally published Figure 3 is shown here for reference:

**Figure 2:**
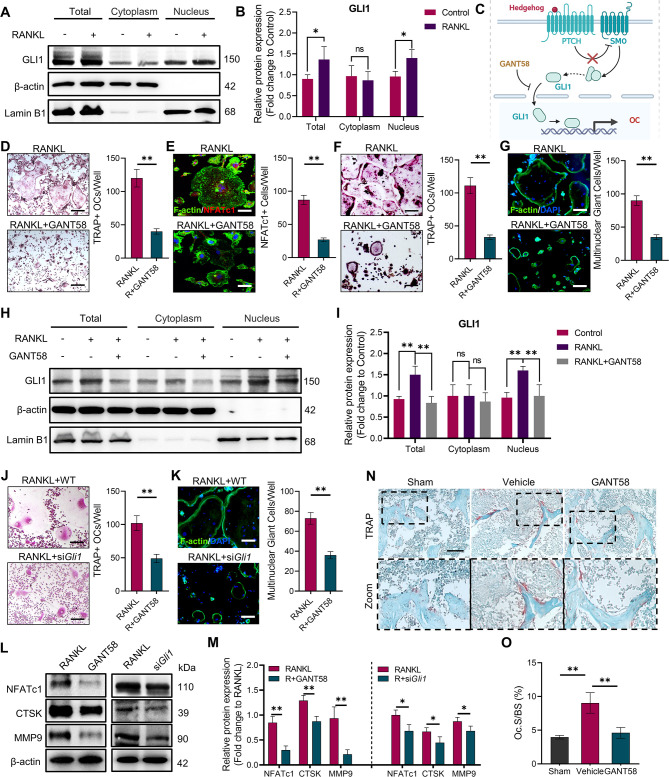


The reviewers approved the new images in corrected Figure 3. The article has been corrected accordingly.

