# Supplementary figures and images for: GLI1 facilitates collagen-induced arthritis in mice by collaborative regulation of DNA methyltransferases
Source: eLife. 2023 Nov 6;12:e92142. doi: 10.7554/eLife.92142 (PMC10627516; doi:10.7554/eLife.92142)

## Slide 1
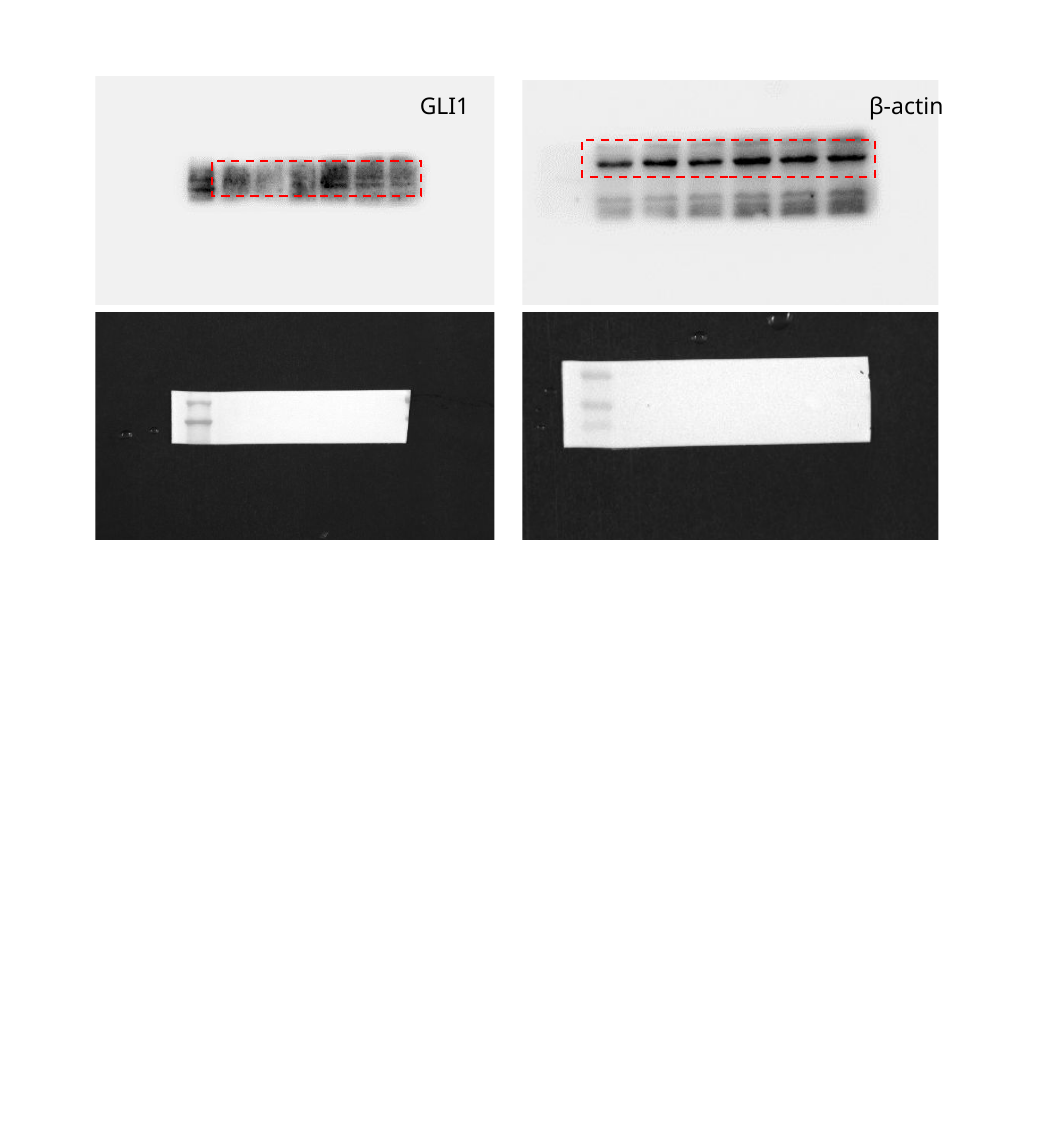

GLI1
β-actin

Supplement: Figure 1—figure supplement 1—source data 1. [file elife-92142-fig1-figsupp1-data1.pptx]

## Slide 1
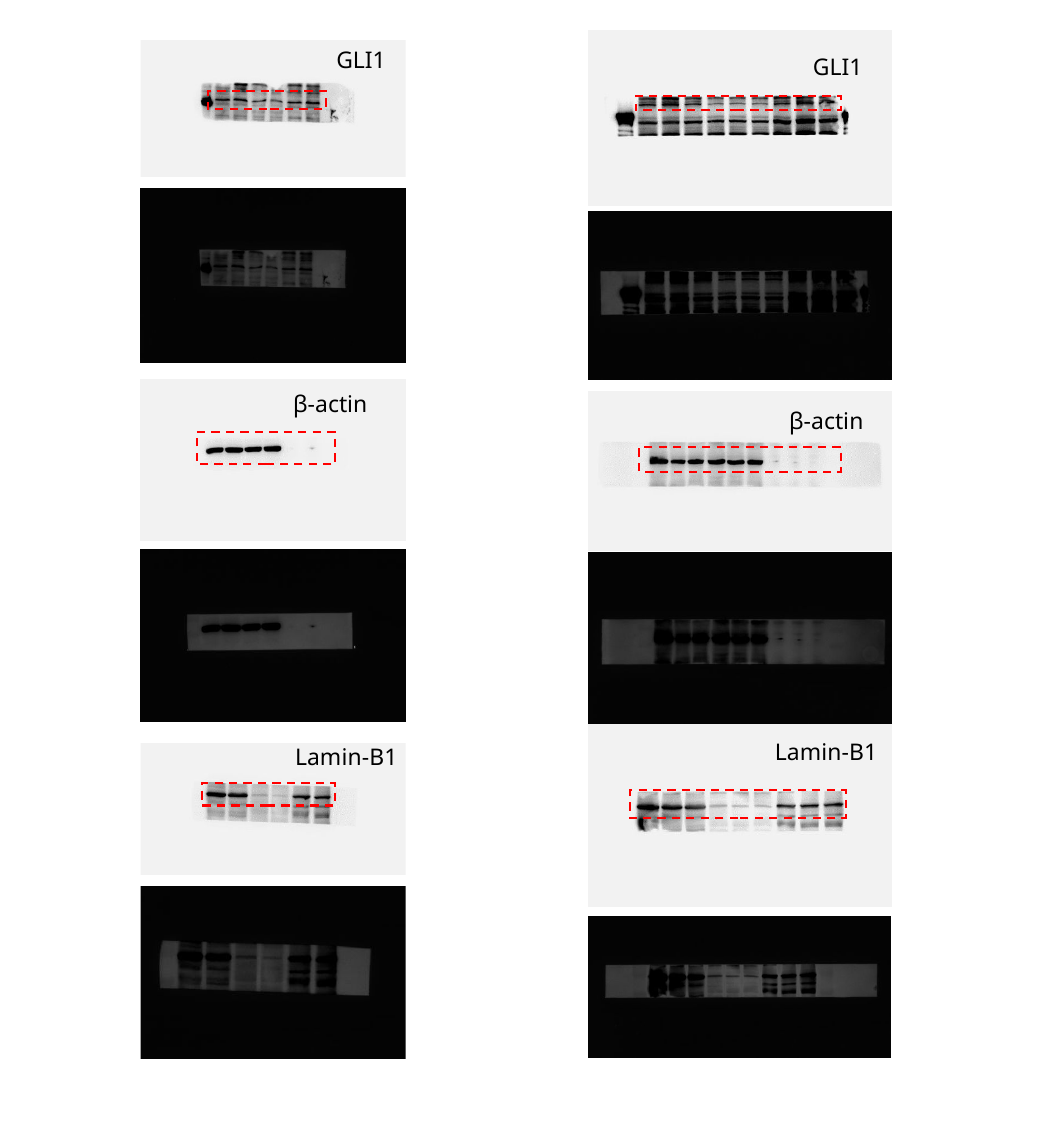

GLI1
GLI1
β-actin
β-actin
Lamin-B1
Lamin-B1

Supplement: Figure 2—source data 1. [file elife-92142-fig2-data1.pptx]

## Slide 1
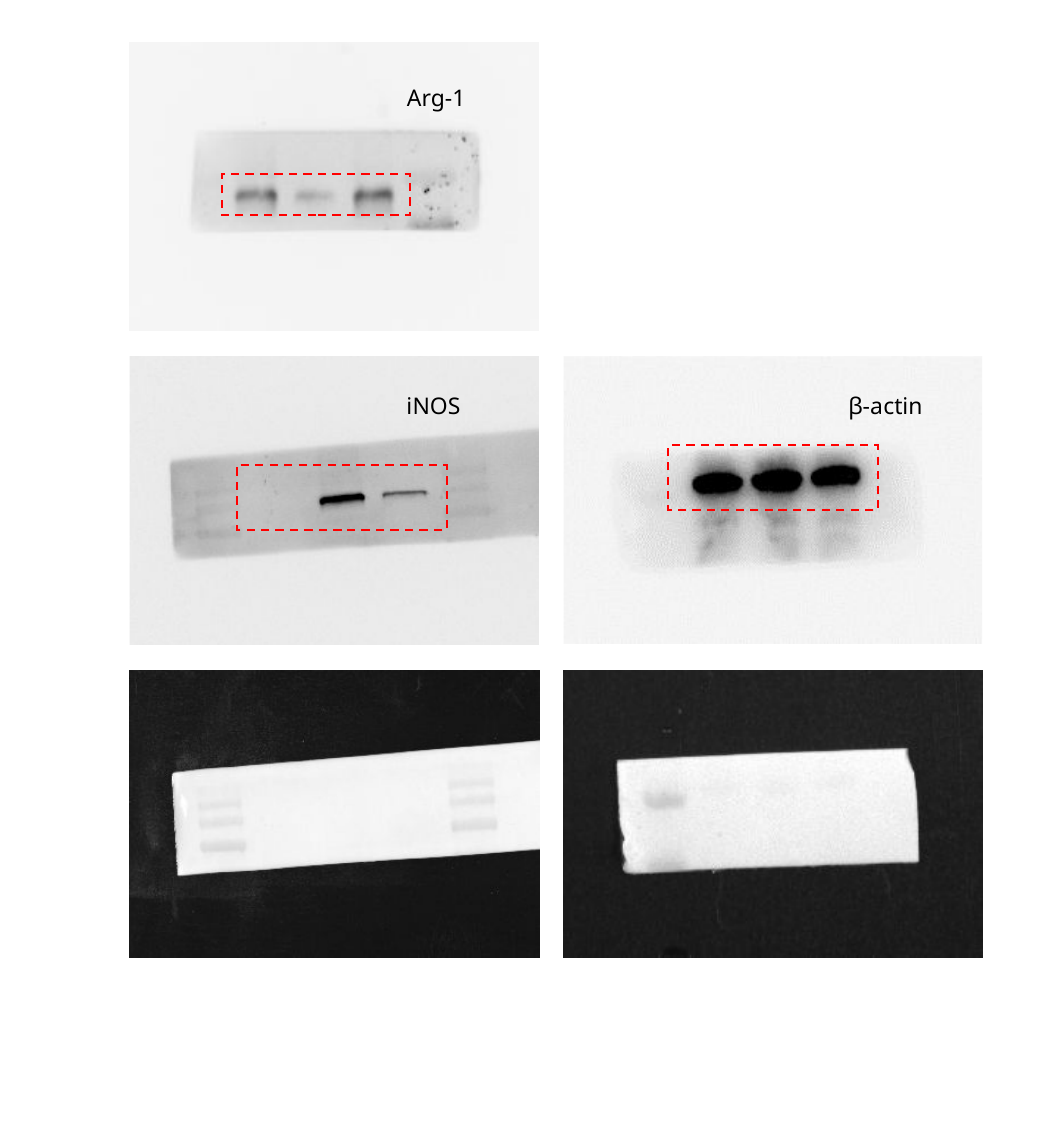

Arg-1
iNOS
β-actin

Supplement: Figure 2—figure supplement 2—source data 1. [file elife-92142-fig2-figsupp2-data1.pptx]

## Slide 1
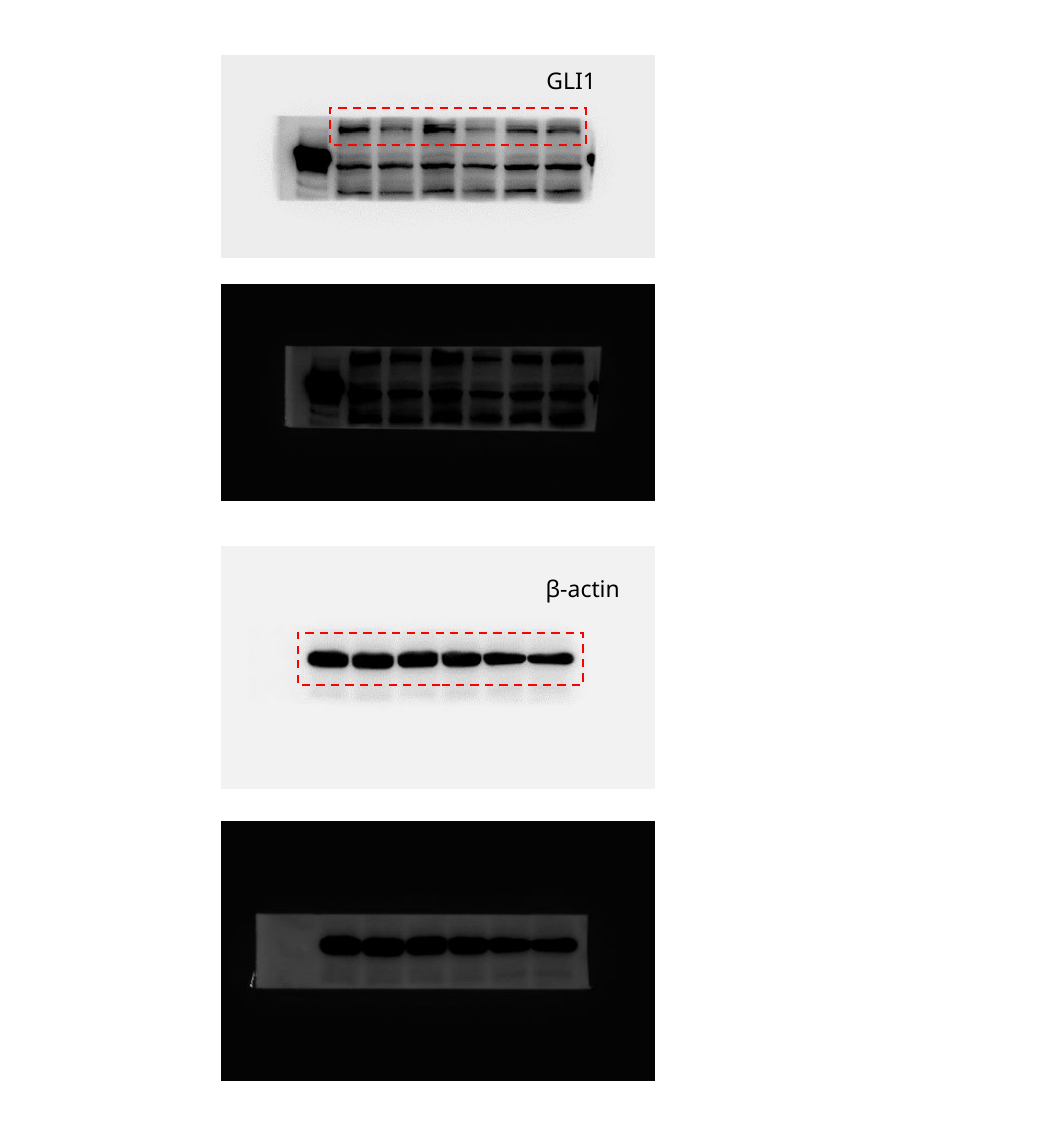

GLI1
β-actin

Supplement: Figure 2—figure supplement 3—source data 1. [file elife-92142-fig2-figsupp3-data1.pptx]

## Slide 1
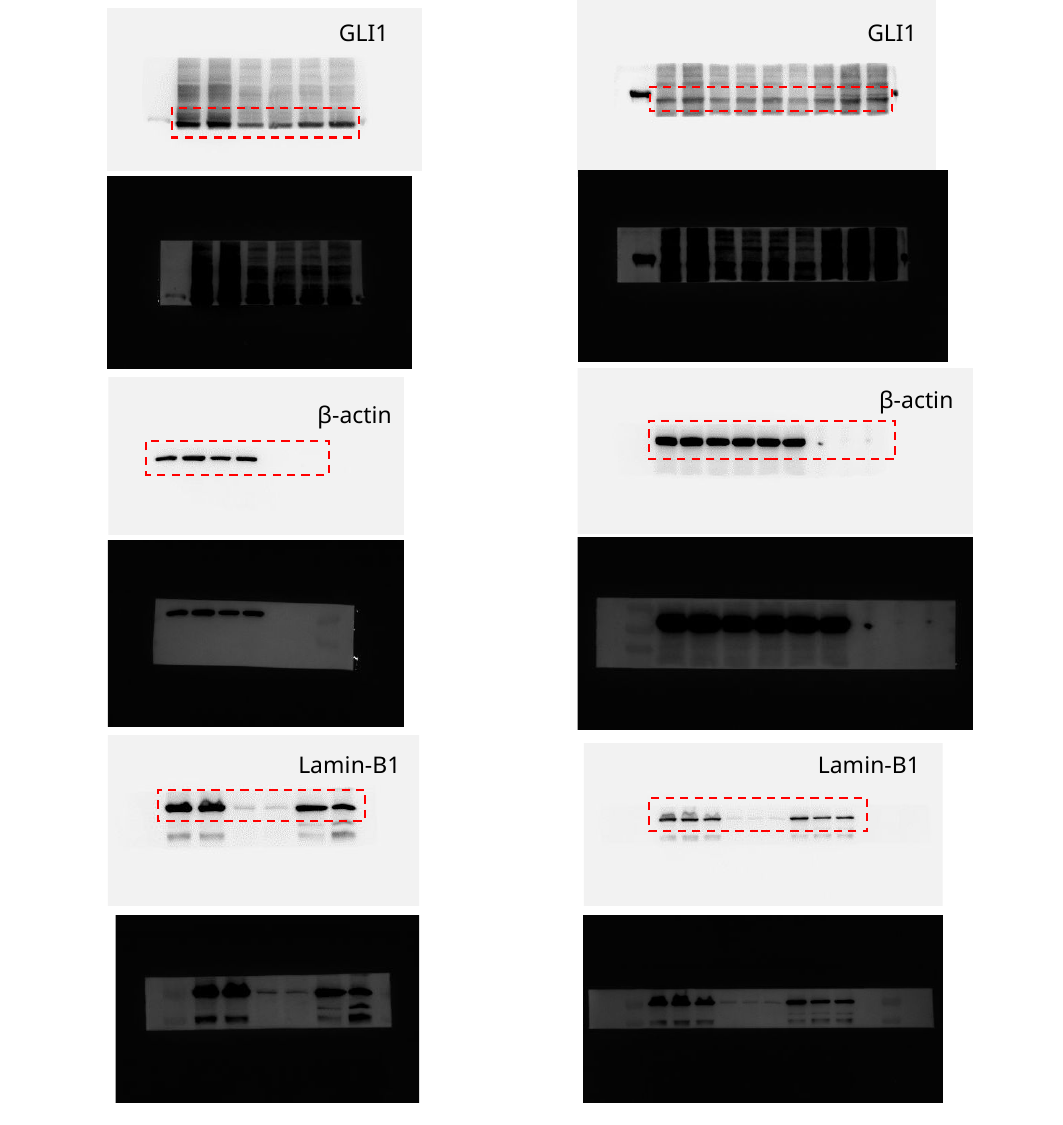

GLI1
GLI1
β-actin
β-actin
Lamin-B1
Lamin-B1

## Slide 2
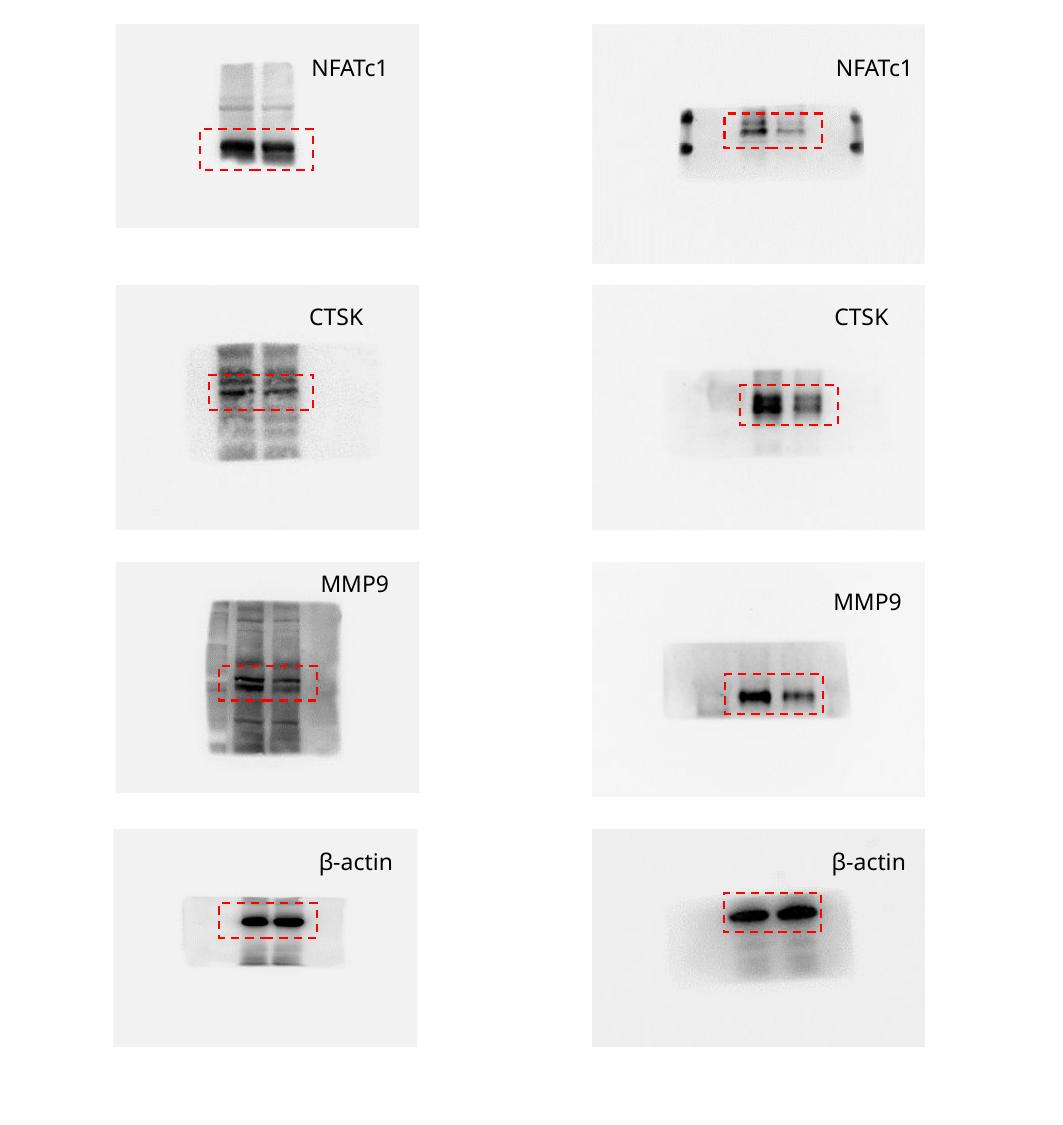

NFATc1
NFATc1
CTSK
CTSK
MMP9
MMP9
β-actin
β-actin

Supplement: Figure 3—source data 1. [file elife-92142-fig3-data1.pptx]

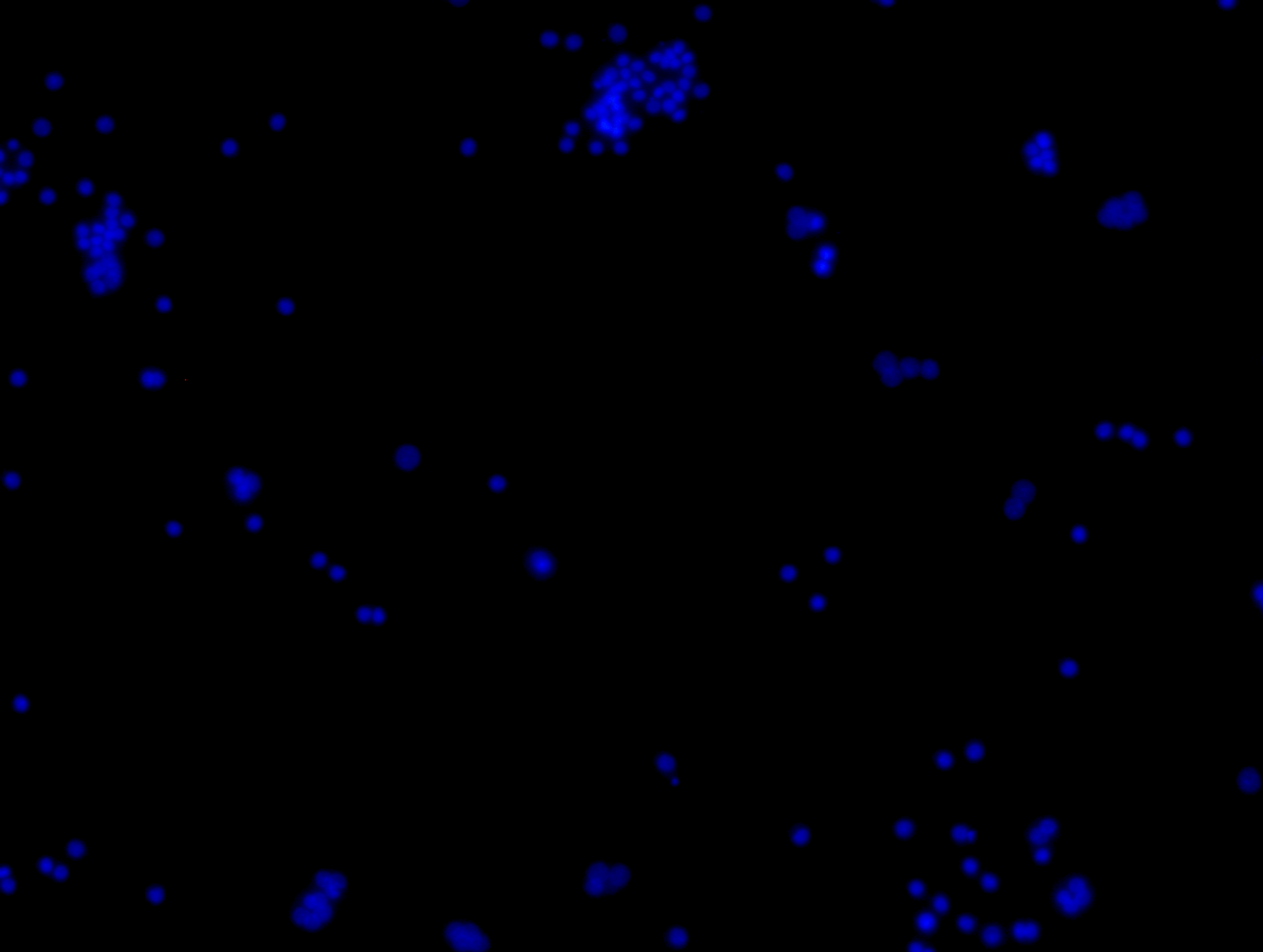

Supplement: Figure 3—source data 2. [file elife-92142-fig3-data2.zip › Source data 2-The raw microscopy images for Figure 3/Figure 3K/RANKL+siGli1 (1).tif]

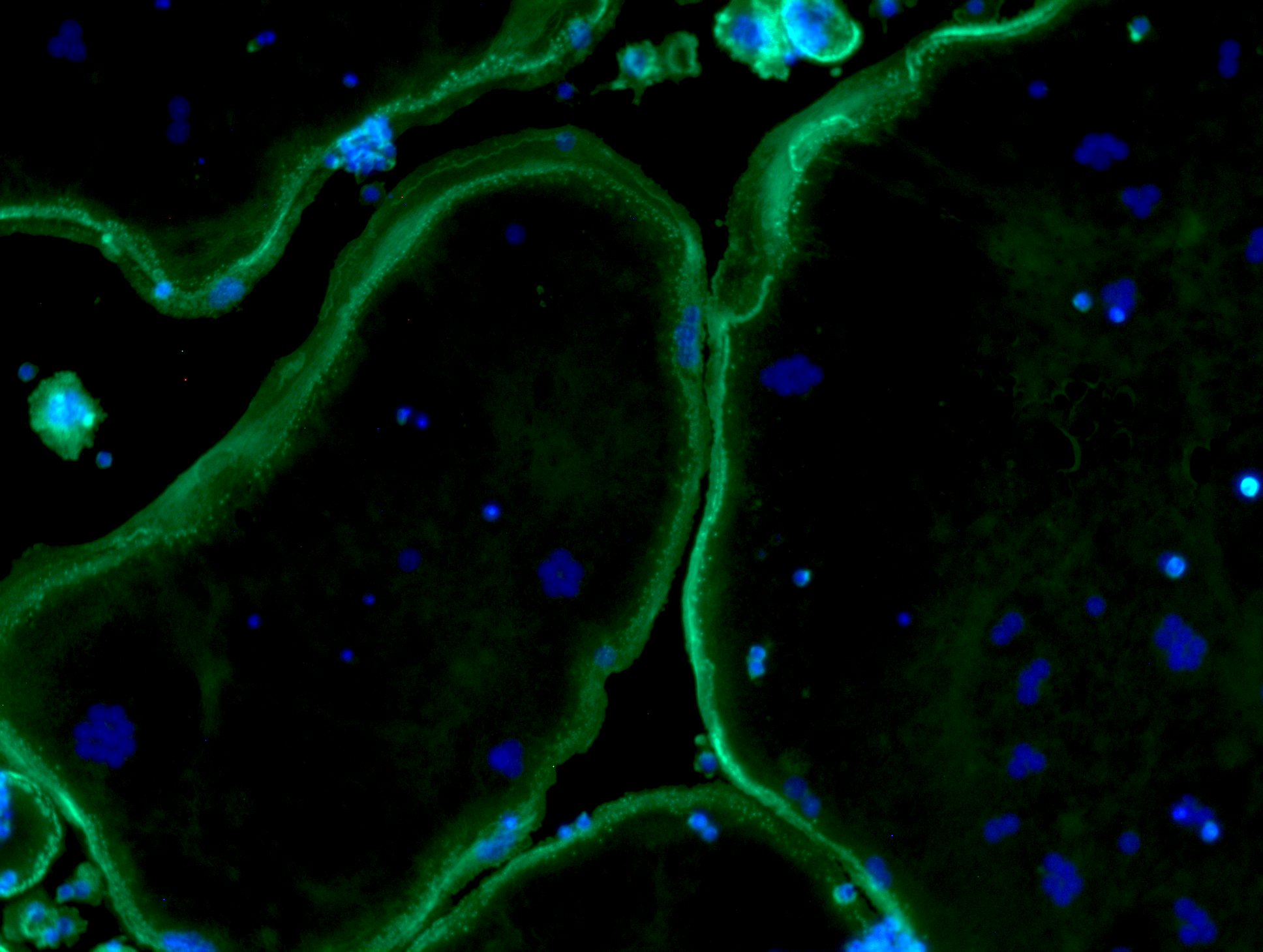

Supplement: Figure 3—source data 2. [file elife-92142-fig3-data2.zip › Source data 2-The raw microscopy images for Figure 3/Figure 3K/RANKL (1).tif]

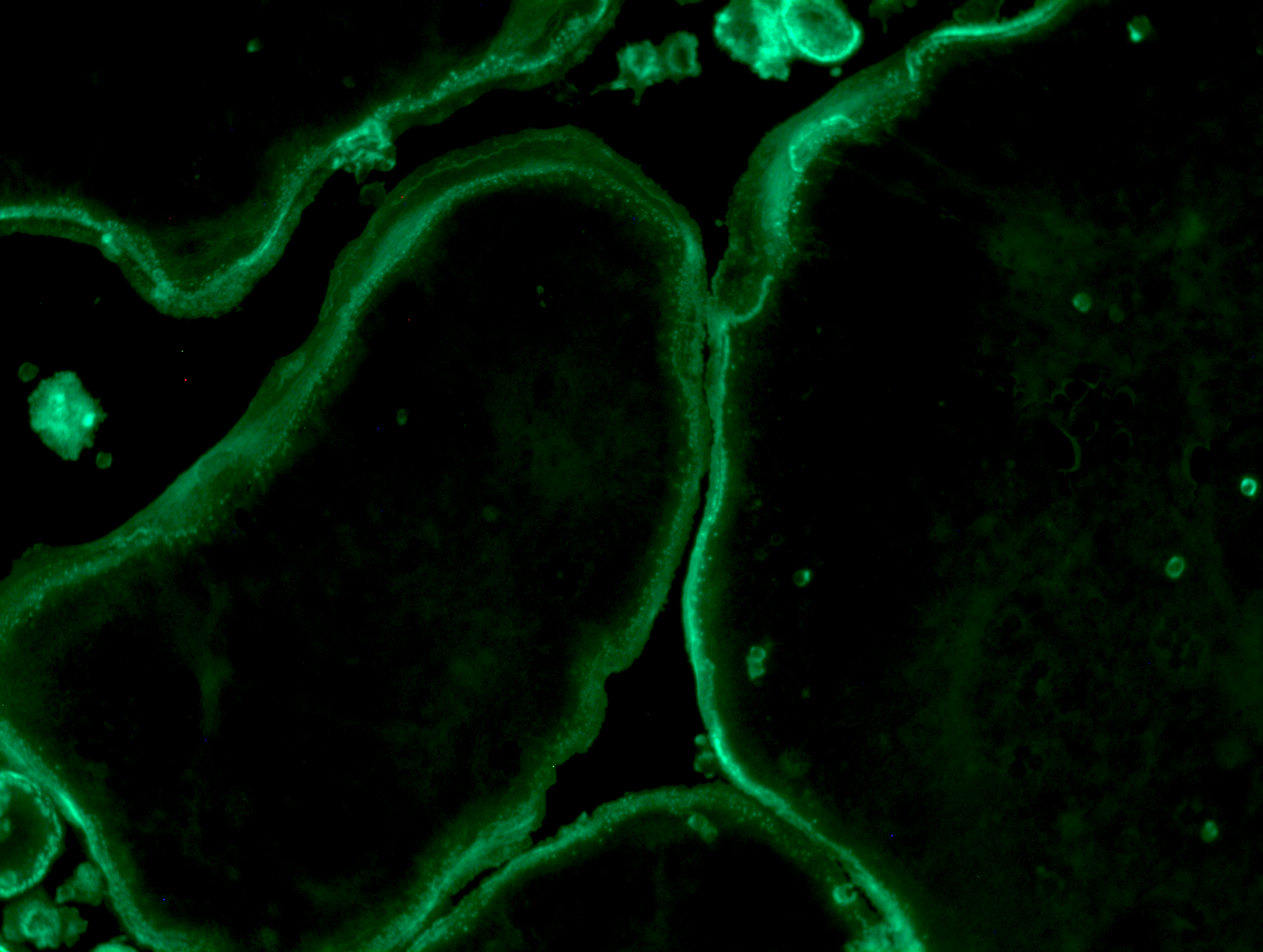

Supplement: Figure 3—source data 2. [file elife-92142-fig3-data2.zip › Source data 2-The raw microscopy images for Figure 3/Figure 3K/RANKL (2).tif]

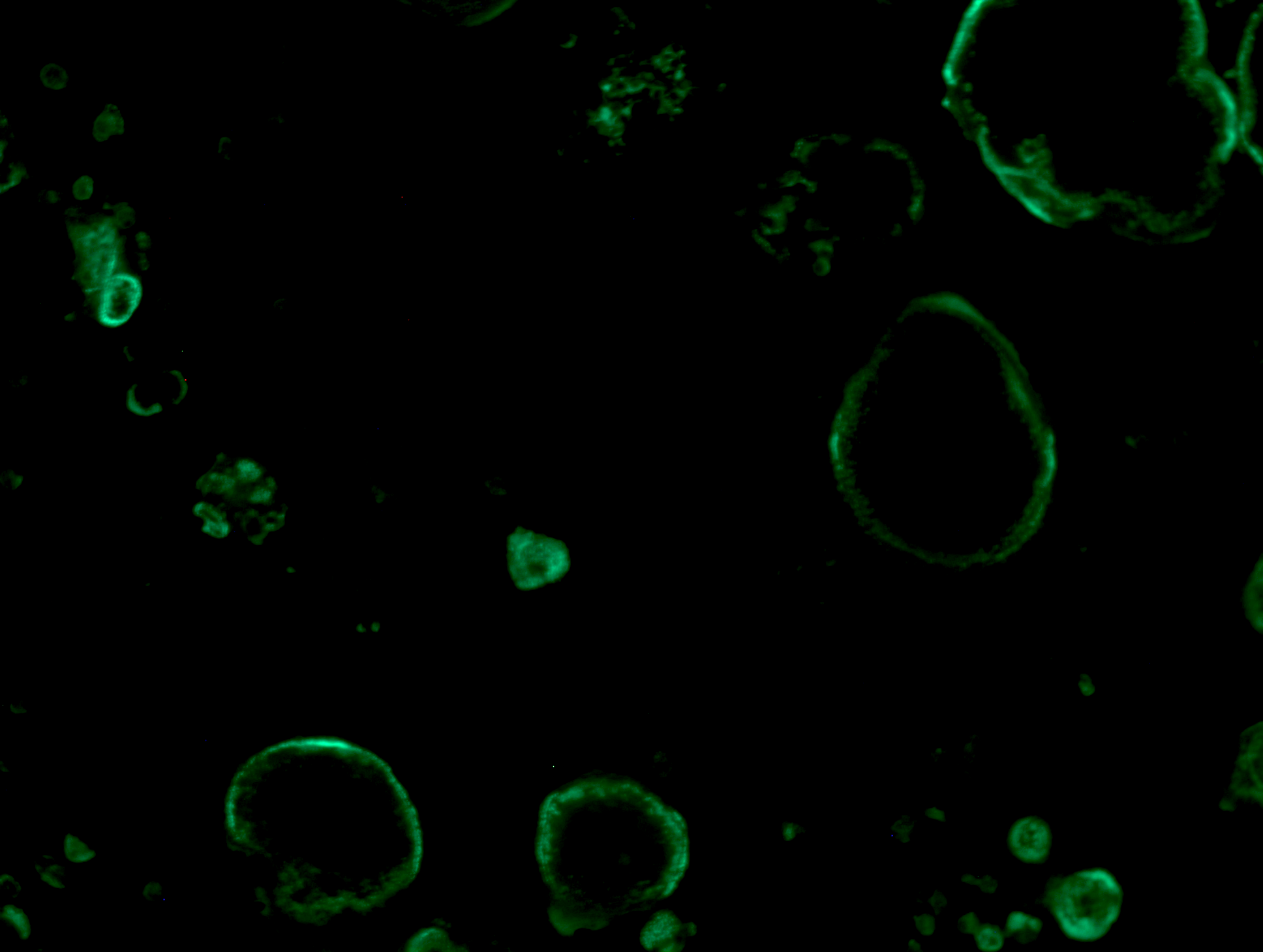

Supplement: Figure 3—source data 2. [file elife-92142-fig3-data2.zip › Source data 2-The raw microscopy images for Figure 3/Figure 3K/RANKL+siGli1 (3).tif]

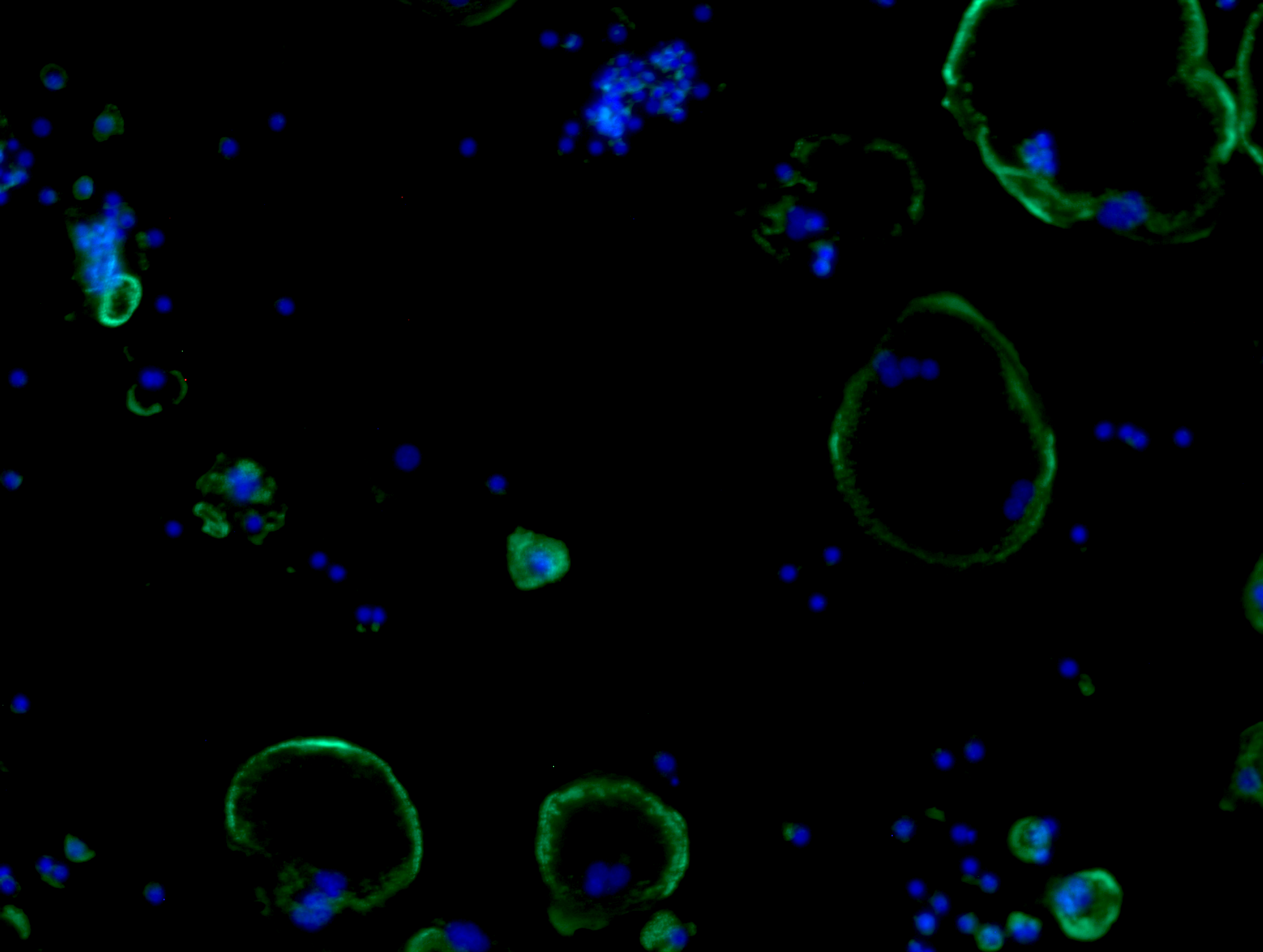

Supplement: Figure 3—source data 2. [file elife-92142-fig3-data2.zip › Source data 2-The raw microscopy images for Figure 3/Figure 3K/RANKL+siGli1 (2).tif]

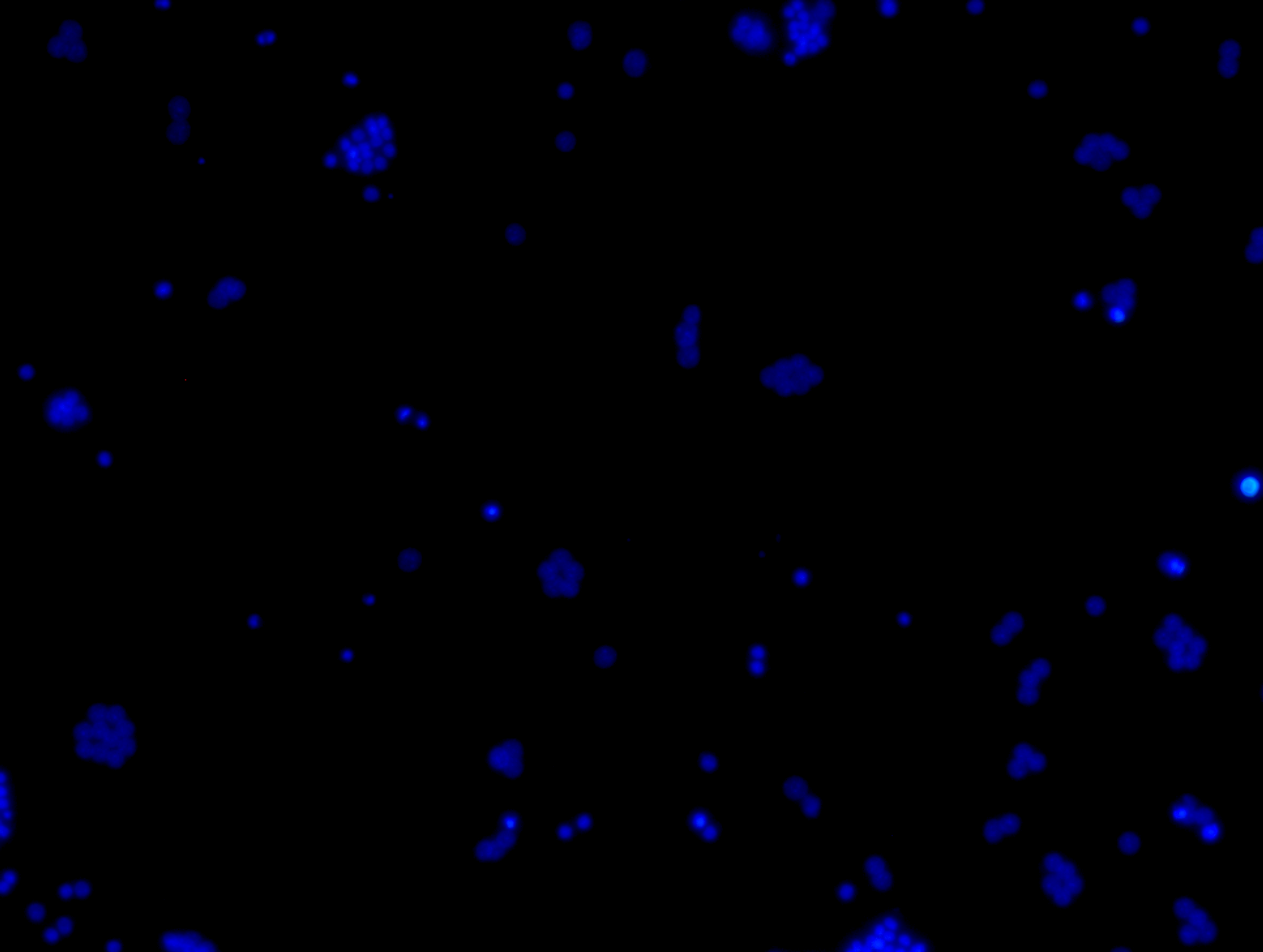

Supplement: Figure 3—source data 2. [file elife-92142-fig3-data2.zip › Source data 2-The raw microscopy images for Figure 3/Figure 3K/RANKL (3).tif]

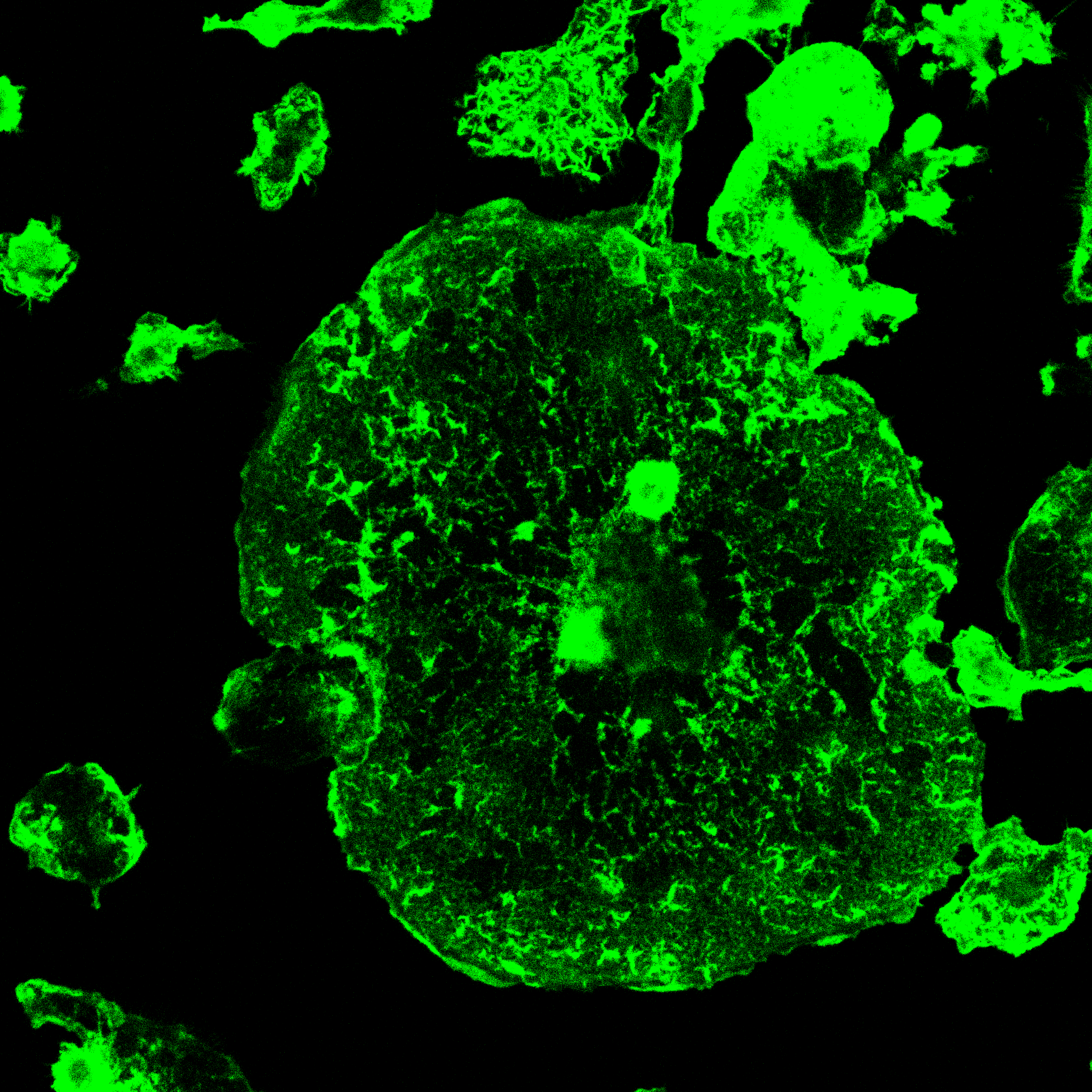

Supplement: Figure 3—source data 2. [file elife-92142-fig3-data2.zip › Source data 2-The raw microscopy images for Figure 3/Figure 3E/BMM NFA RANKL (1).tif]

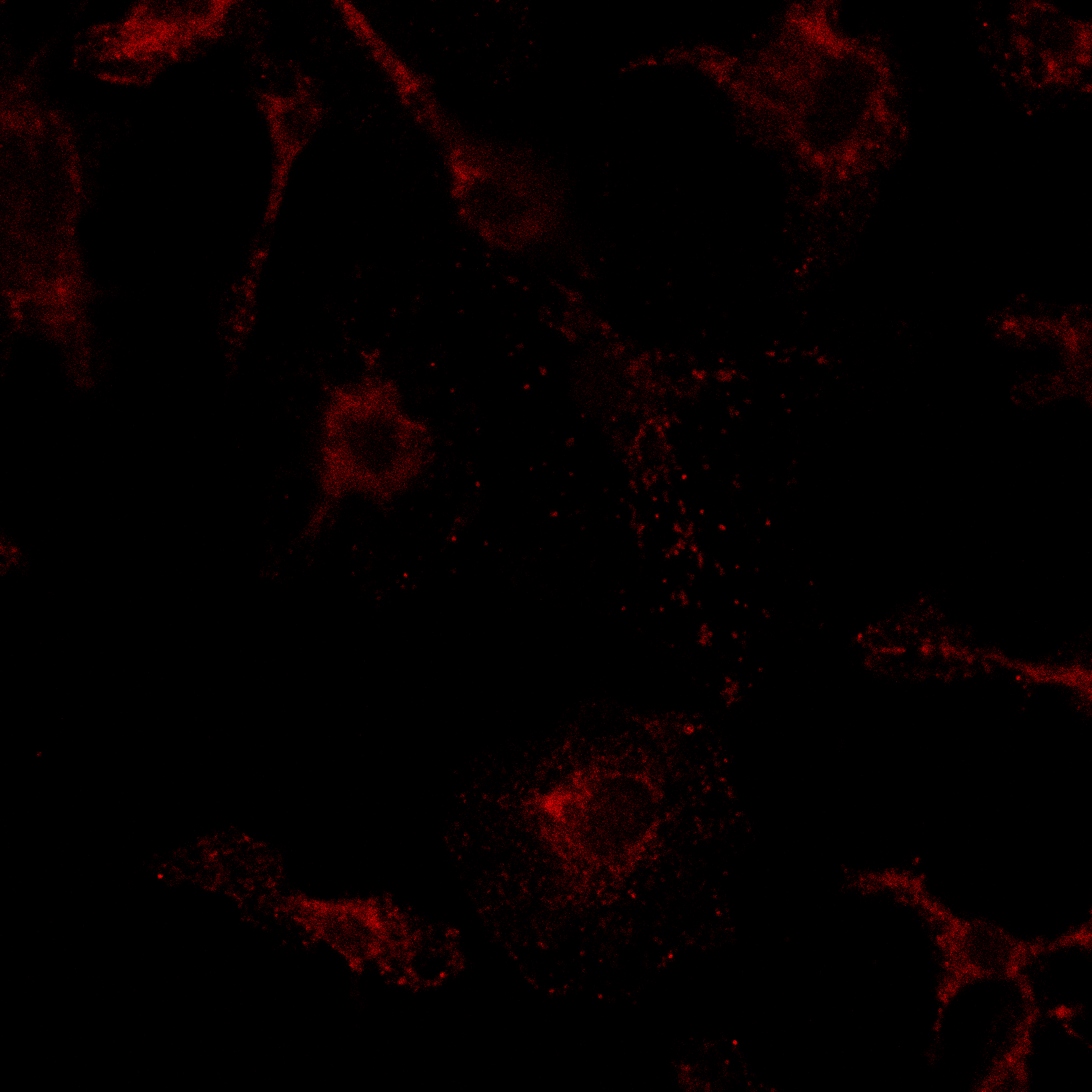

Supplement: Figure 3—source data 2. [file elife-92142-fig3-data2.zip › Source data 2-The raw microscopy images for Figure 3/Figure 3E/BMM NFA GANT58 (4).tif]

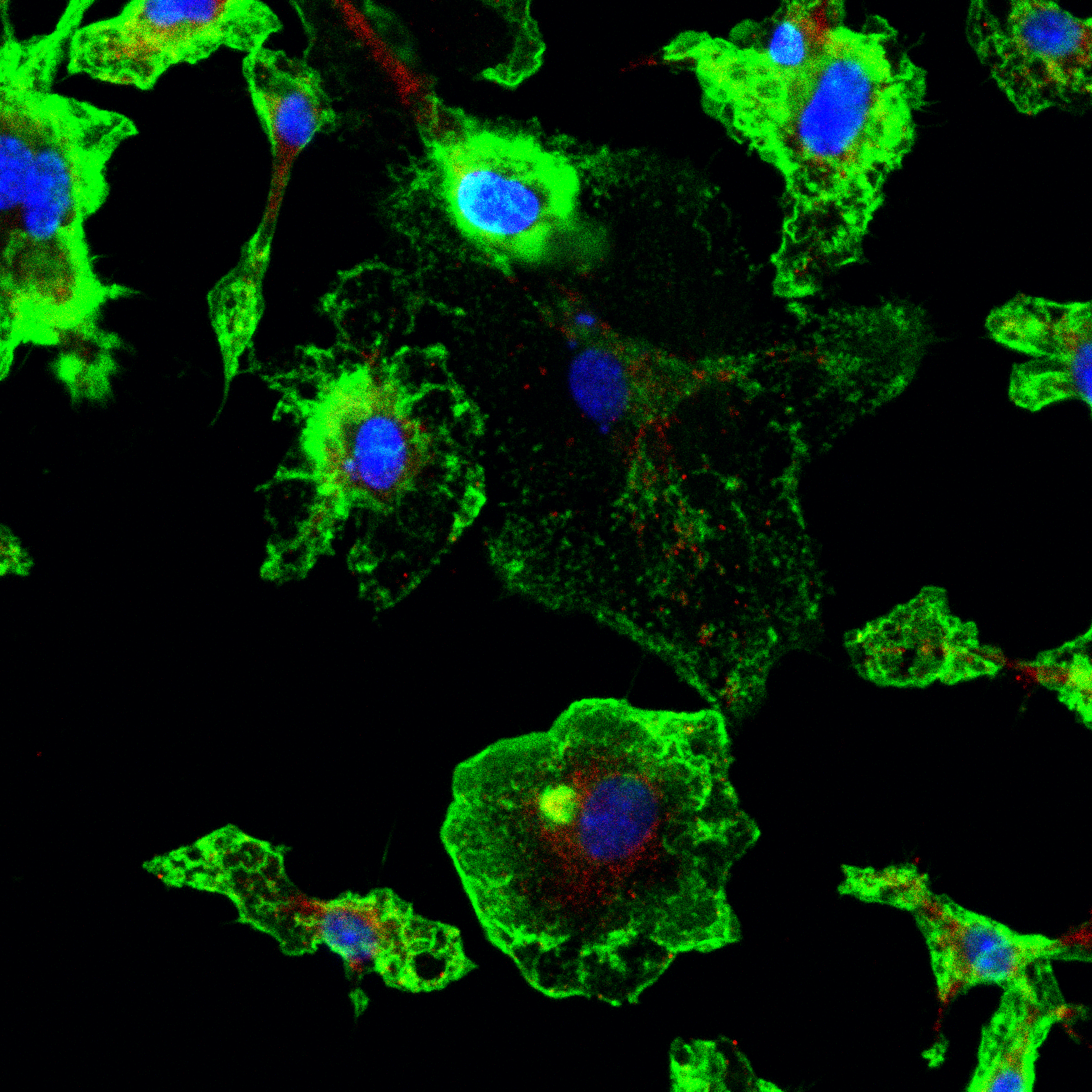

Supplement: Figure 3—source data 2. [file elife-92142-fig3-data2.zip › Source data 2-The raw microscopy images for Figure 3/Figure 3E/BMM NFA GANT58 (2).tif]

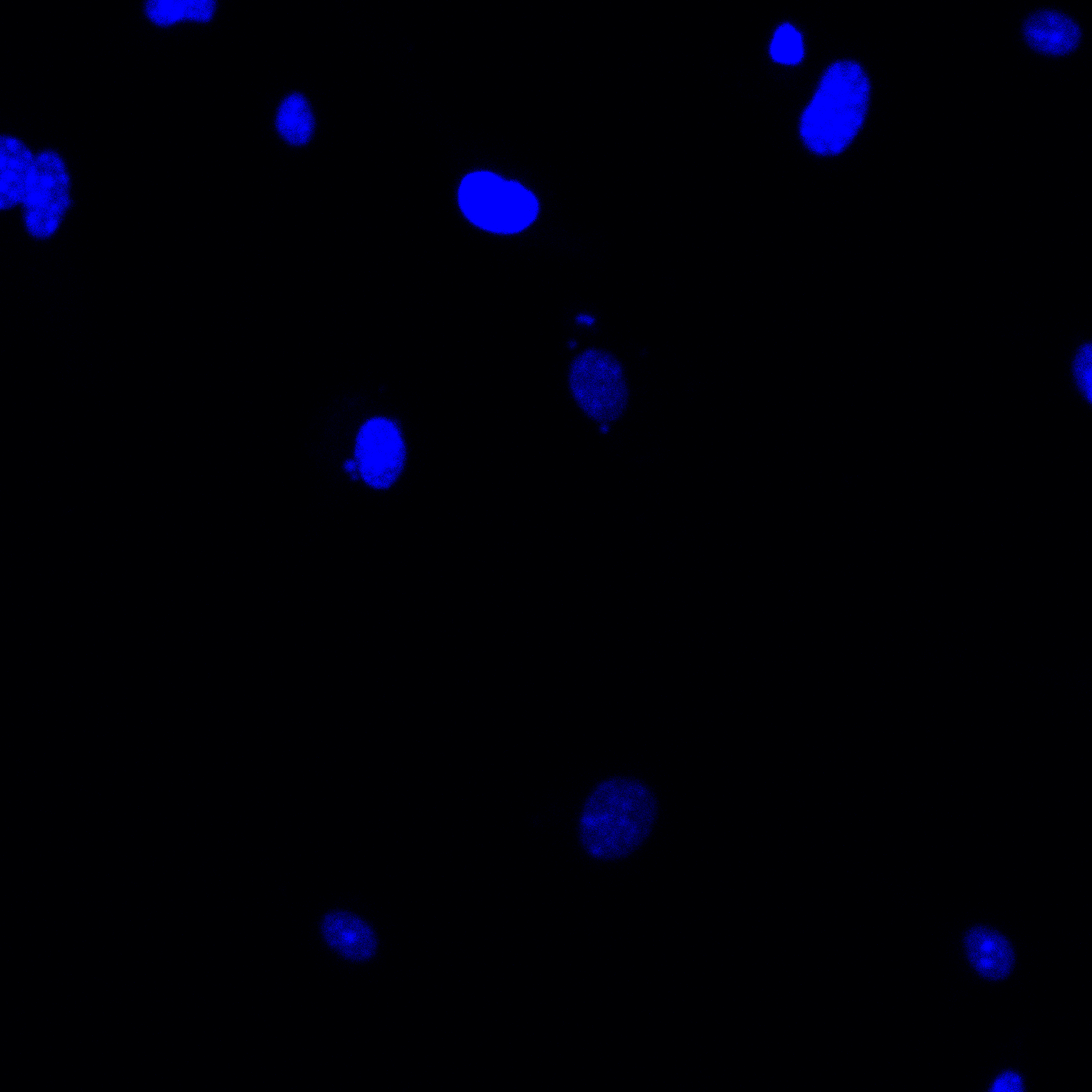

Supplement: Figure 3—source data 2. [file elife-92142-fig3-data2.zip › Source data 2-The raw microscopy images for Figure 3/Figure 3E/BMM NFA GANT58 (3).tif]

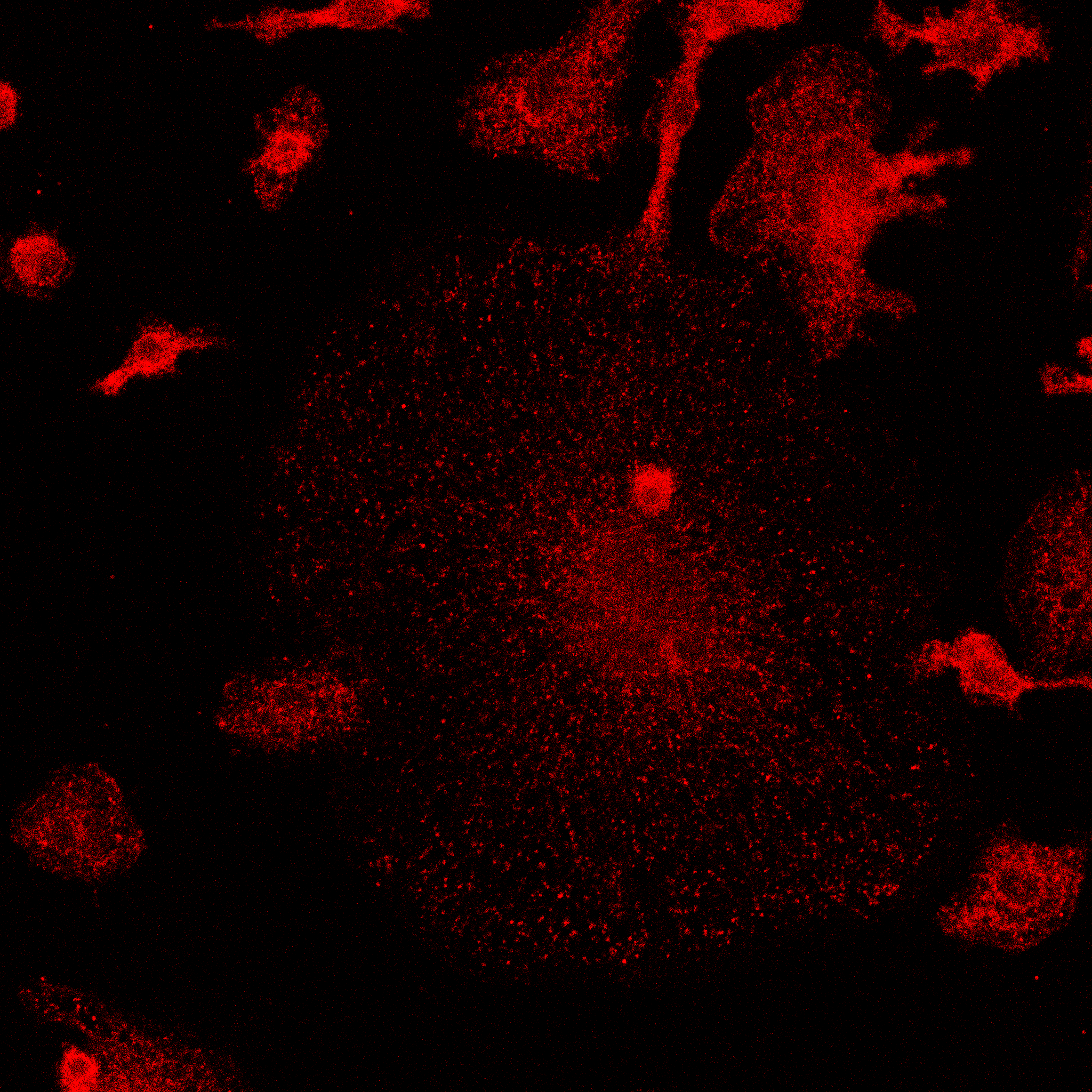

Supplement: Figure 3—source data 2. [file elife-92142-fig3-data2.zip › Source data 2-The raw microscopy images for Figure 3/Figure 3E/BMM NFA RANKL (4).tif]

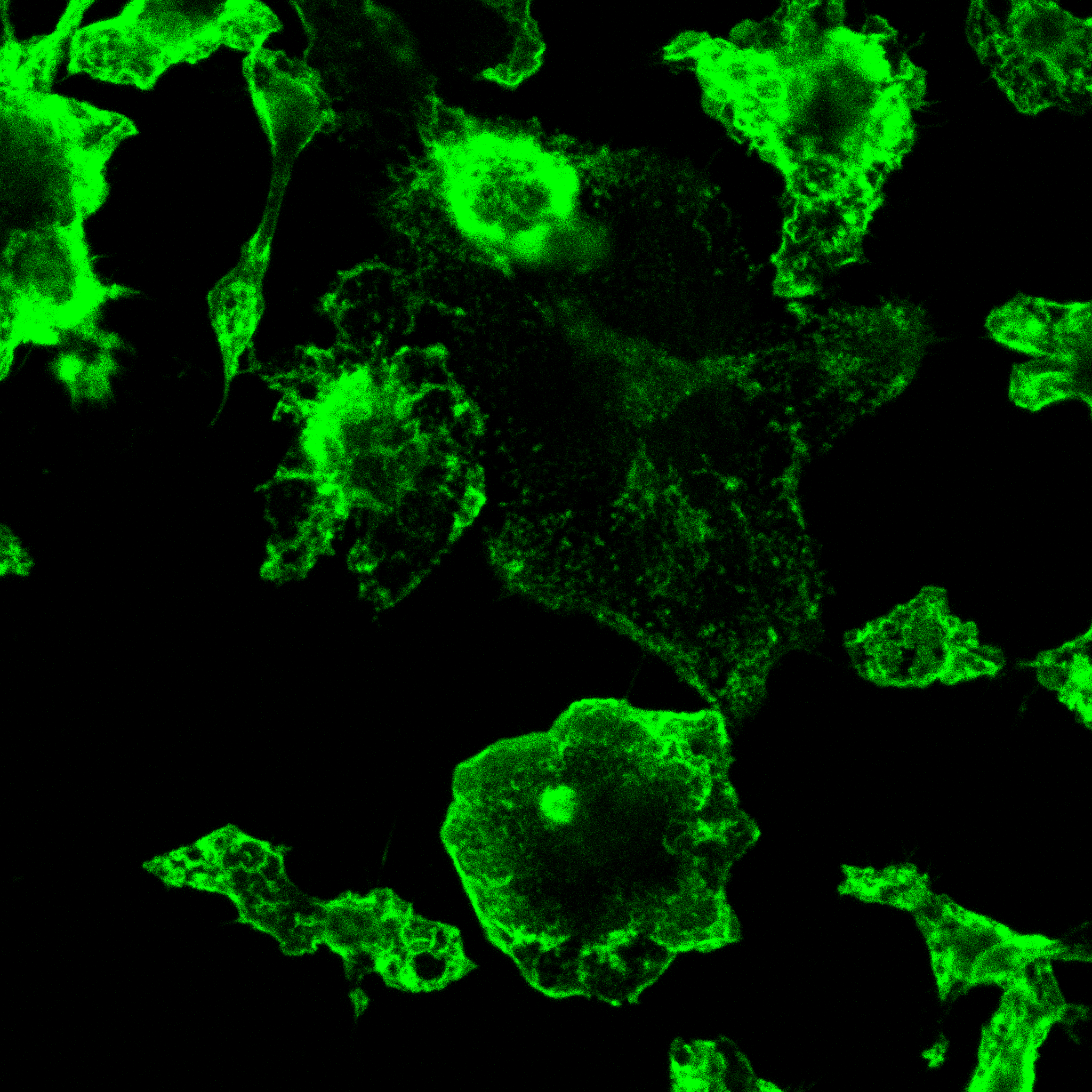

Supplement: Figure 3—source data 2. [file elife-92142-fig3-data2.zip › Source data 2-The raw microscopy images for Figure 3/Figure 3E/BMM NFA GANT58 (1).tif]

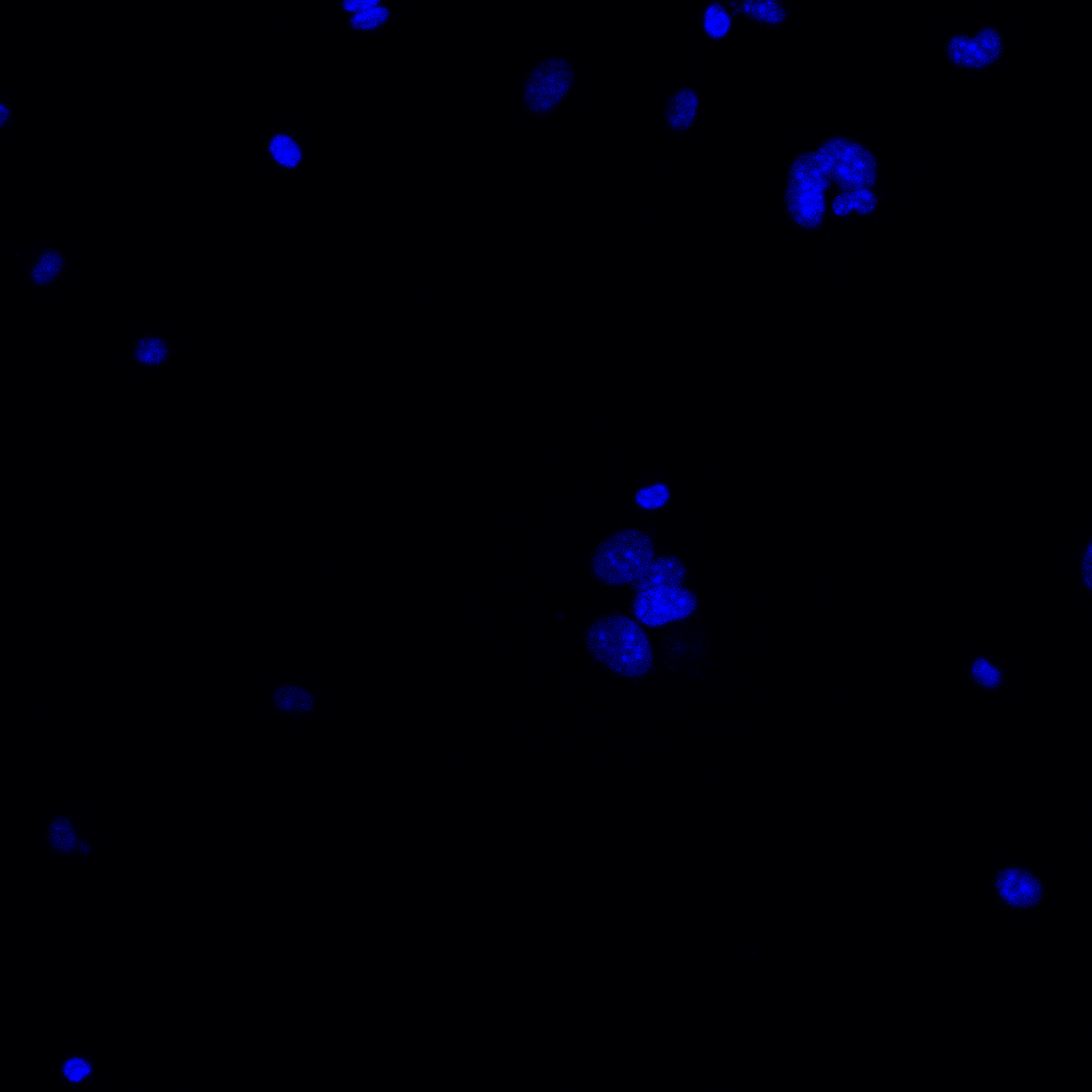

Supplement: Figure 3—source data 2. [file elife-92142-fig3-data2.zip › Source data 2-The raw microscopy images for Figure 3/Figure 3E/BMM NFA RANKL (3).tif]

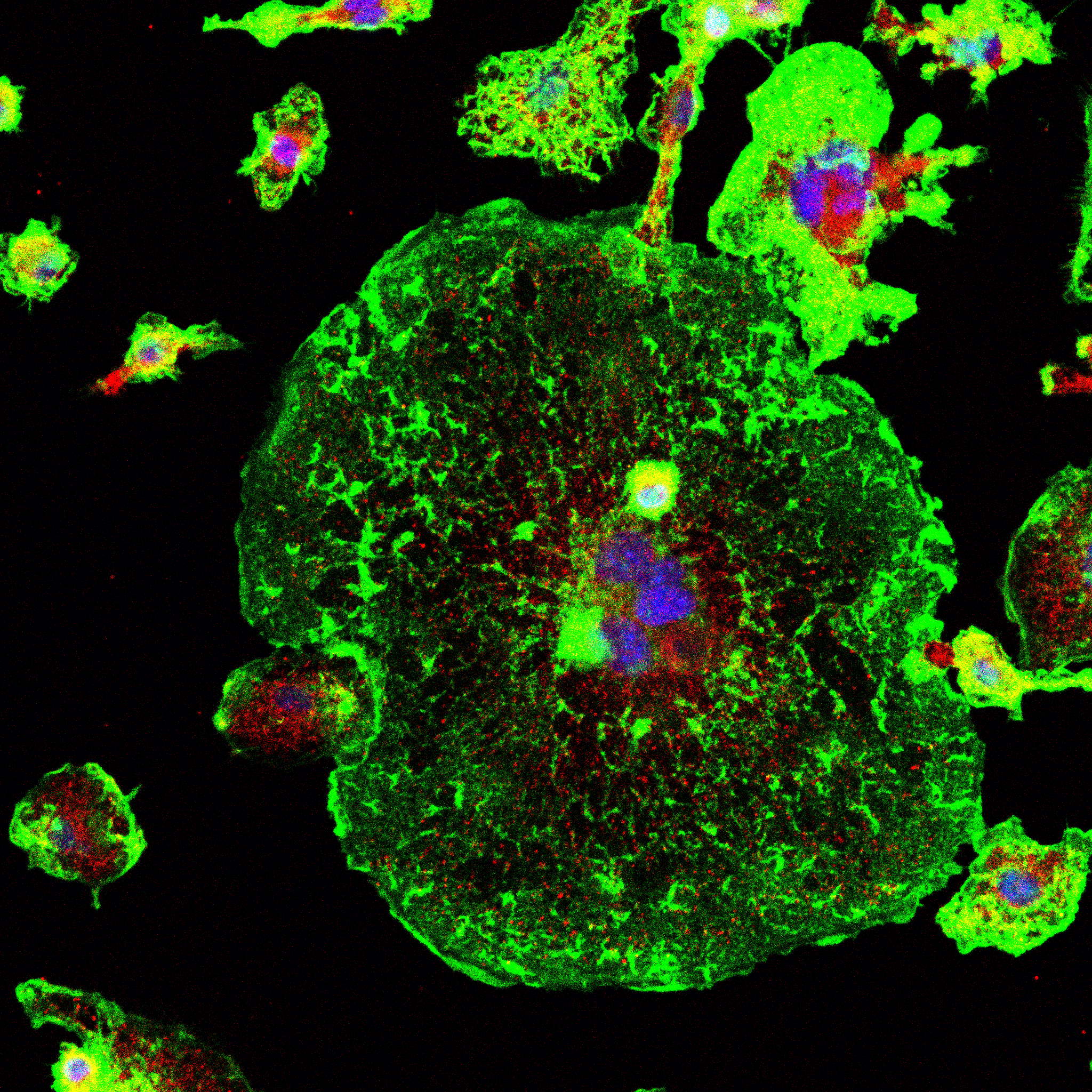

Supplement: Figure 3—source data 2. [file elife-92142-fig3-data2.zip › Source data 2-The raw microscopy images for Figure 3/Figure 3E/BMM NFA RANKL (2).tif]

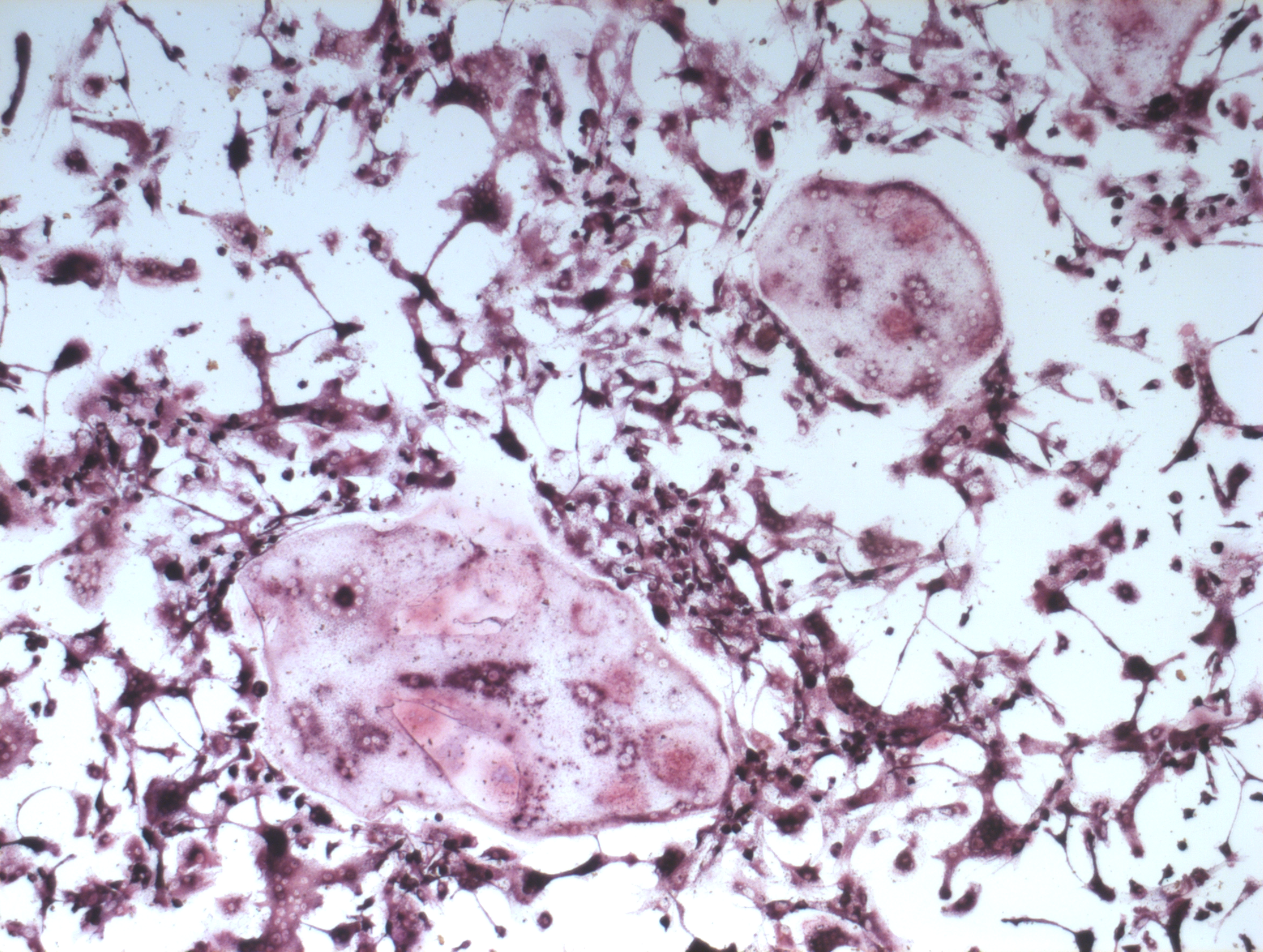

Supplement: Figure 3—source data 2. [file elife-92142-fig3-data2.zip › Source data 2-The raw microscopy images for Figure 3/Figure 3D/RANKL.tif]

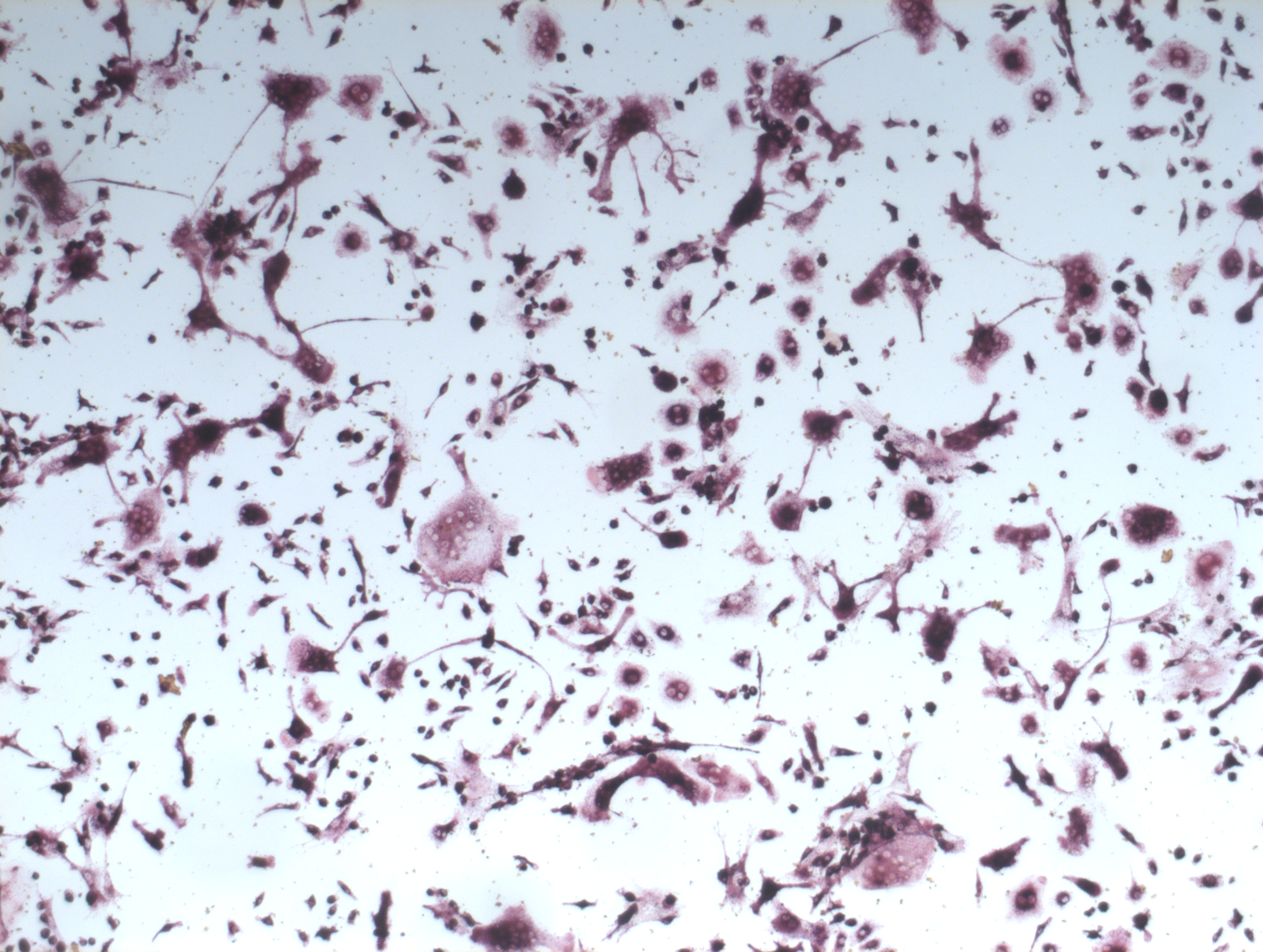

Supplement: Figure 3—source data 2. [file elife-92142-fig3-data2.zip › Source data 2-The raw microscopy images for Figure 3/Figure 3D/RANKL GANT58.tif]

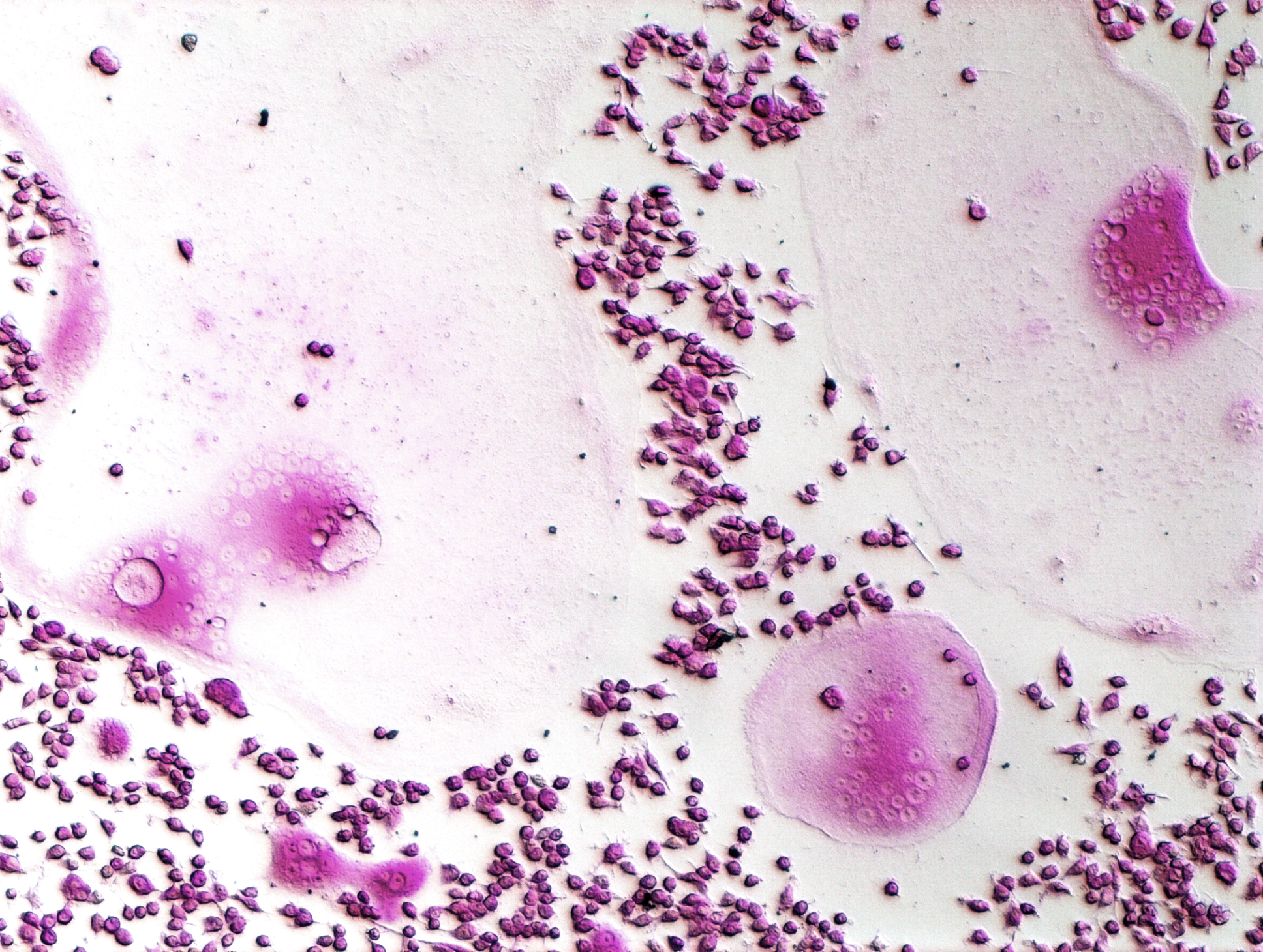

Supplement: Figure 3—source data 2. [file elife-92142-fig3-data2.zip › Source data 2-The raw microscopy images for Figure 3/Figure 3J/RANKL.tif]

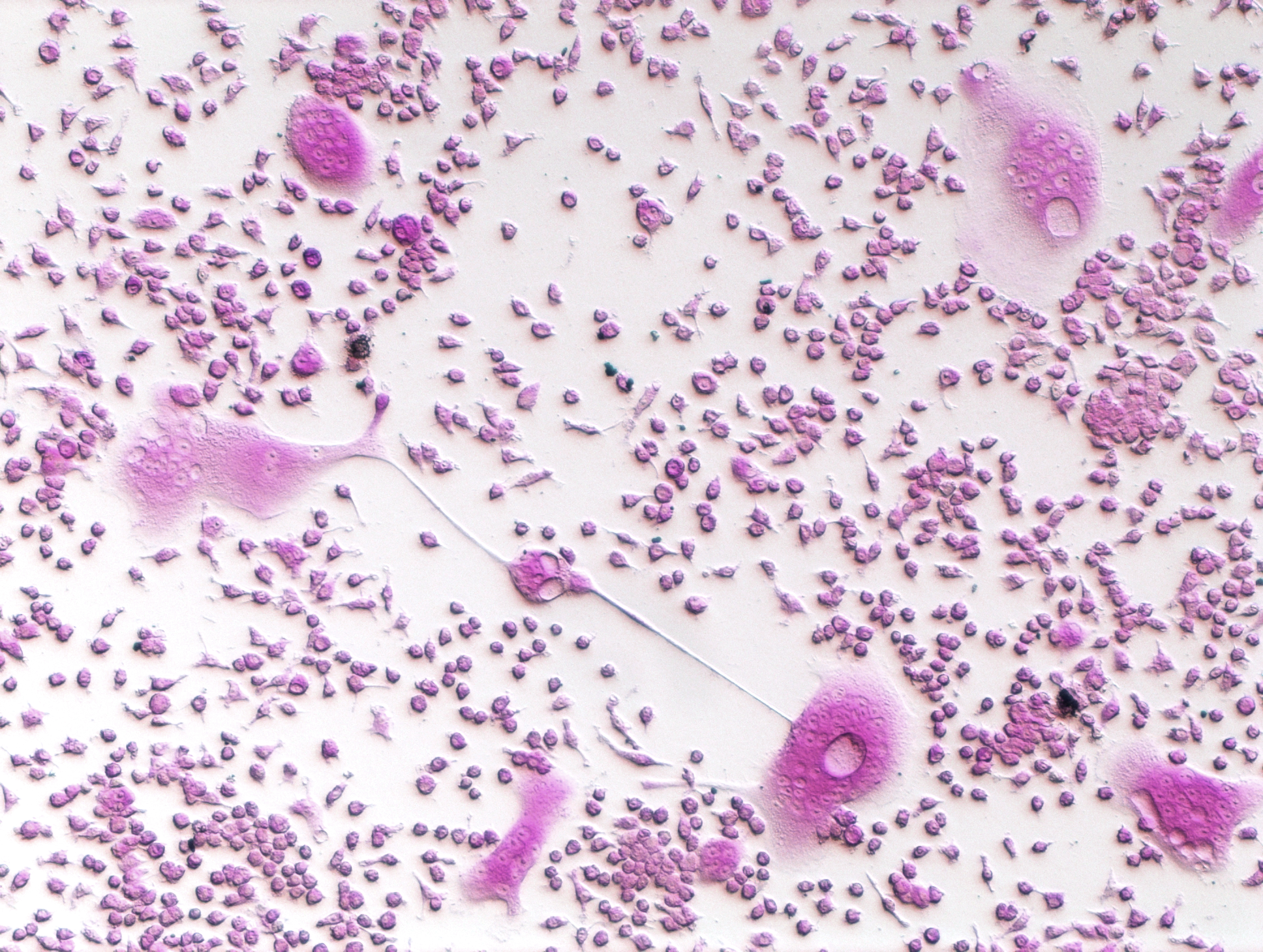

Supplement: Figure 3—source data 2. [file elife-92142-fig3-data2.zip › Source data 2-The raw microscopy images for Figure 3/Figure 3J/RANKL+siGli1.tif]

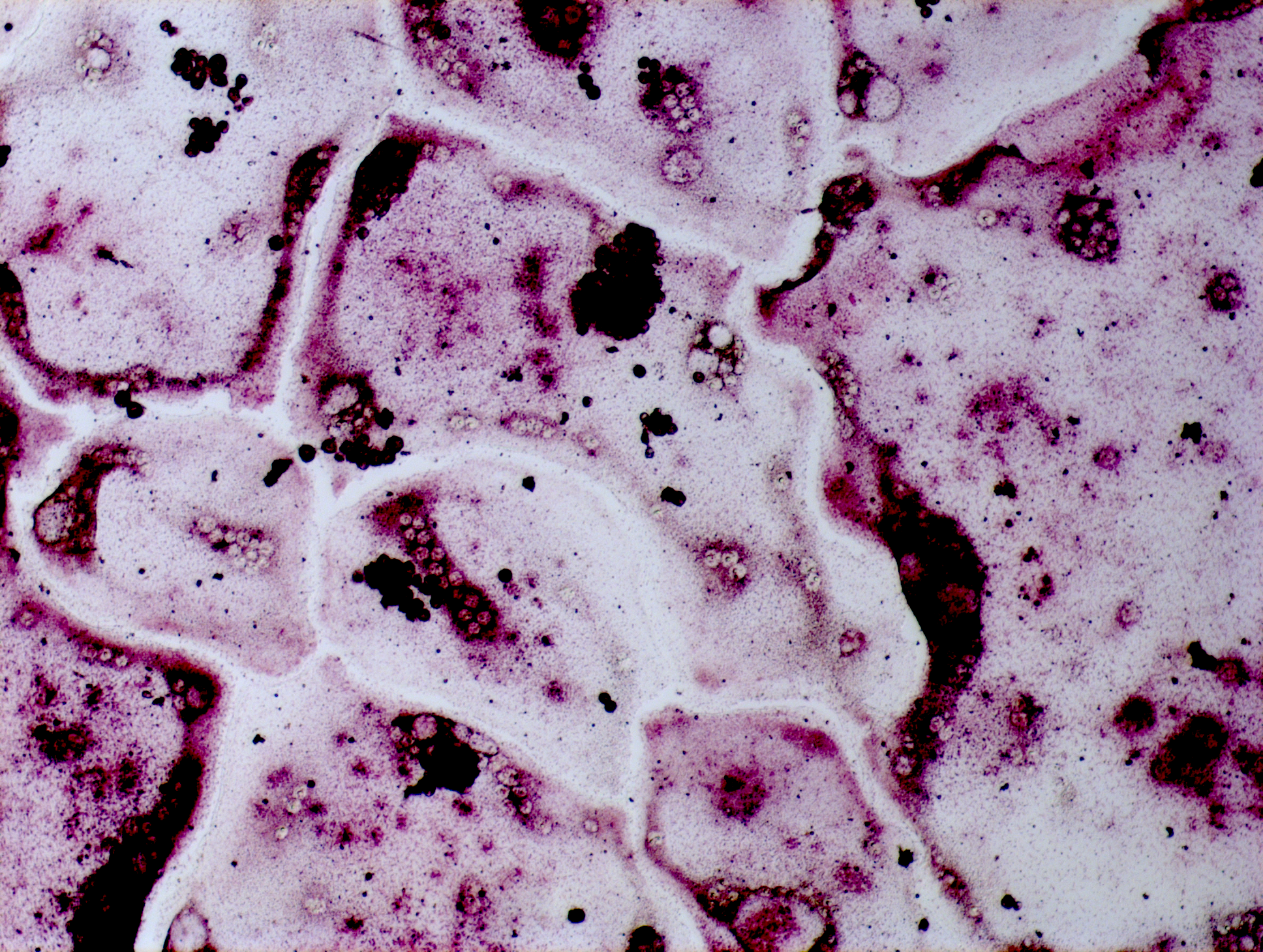

Supplement: Figure 3—source data 2. [file elife-92142-fig3-data2.zip › Source data 2-The raw microscopy images for Figure 3/Figure 3F/RANKL.tif]

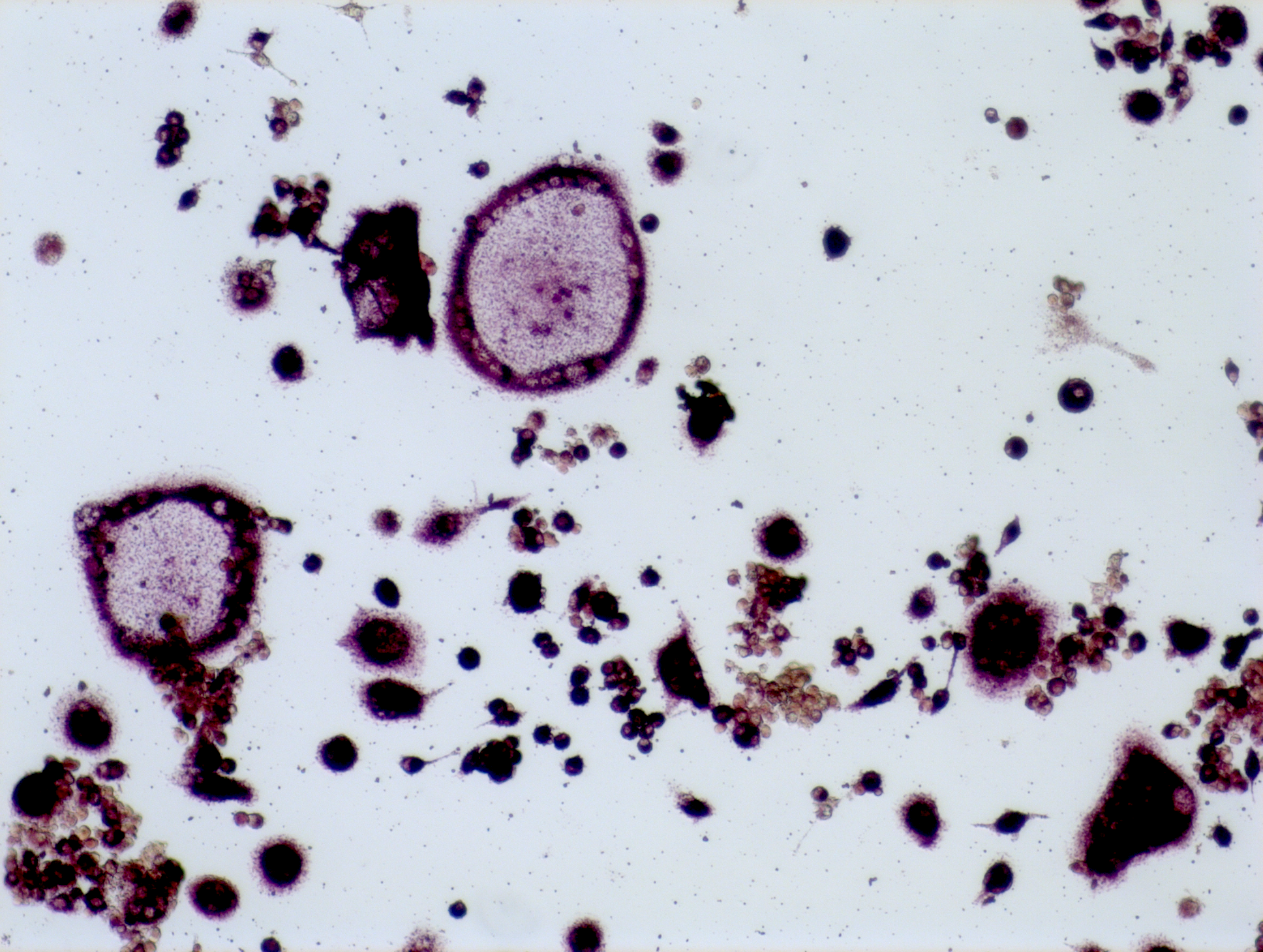

Supplement: Figure 3—source data 2. [file elife-92142-fig3-data2.zip › Source data 2-The raw microscopy images for Figure 3/Figure 3F/RANKL+GANT58.tif]

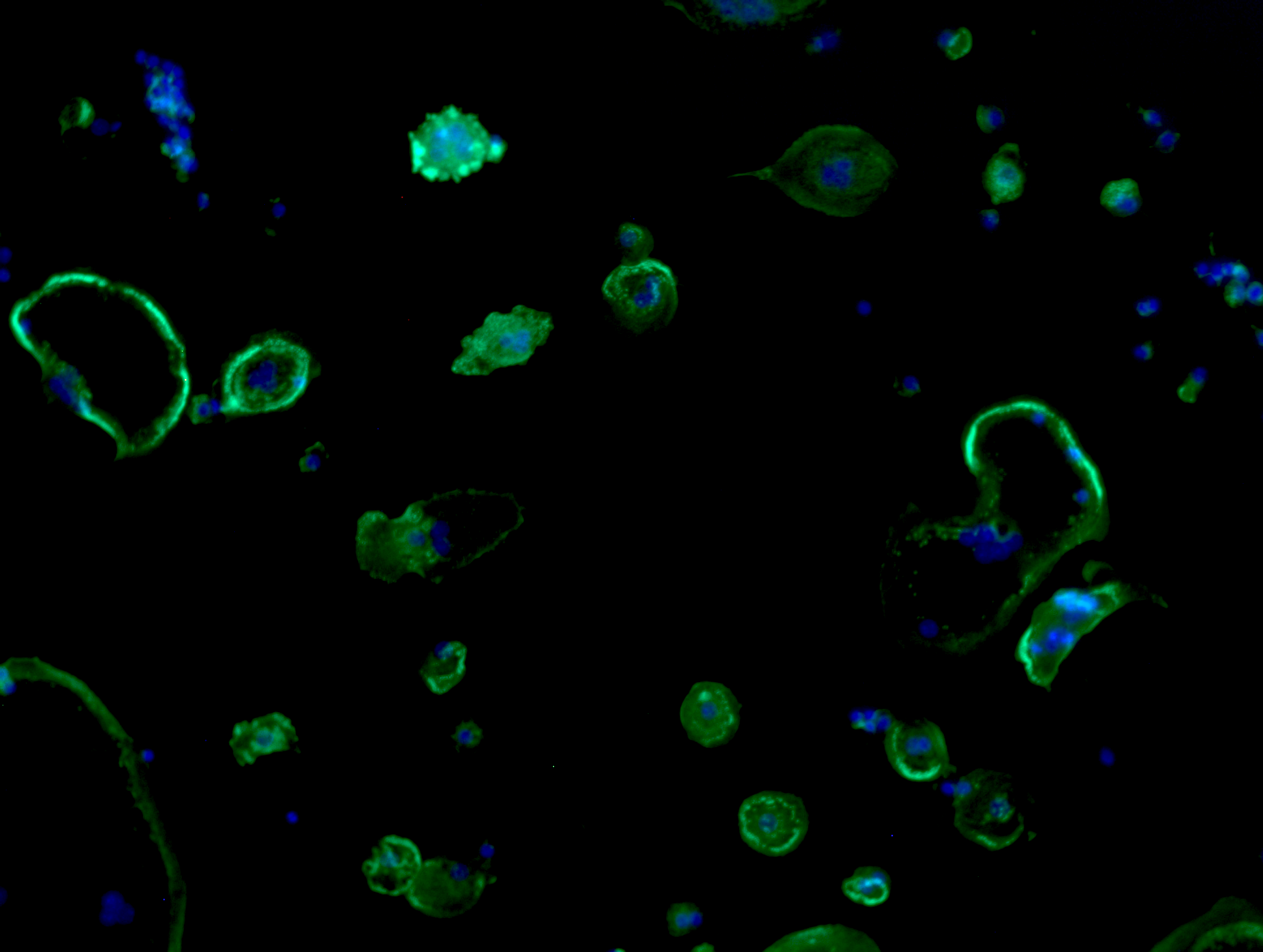

Supplement: Figure 3—source data 2. [file elife-92142-fig3-data2.zip › Source data 2-The raw microscopy images for Figure 3/Figure 3G/RAW GANT58 (2).tif]

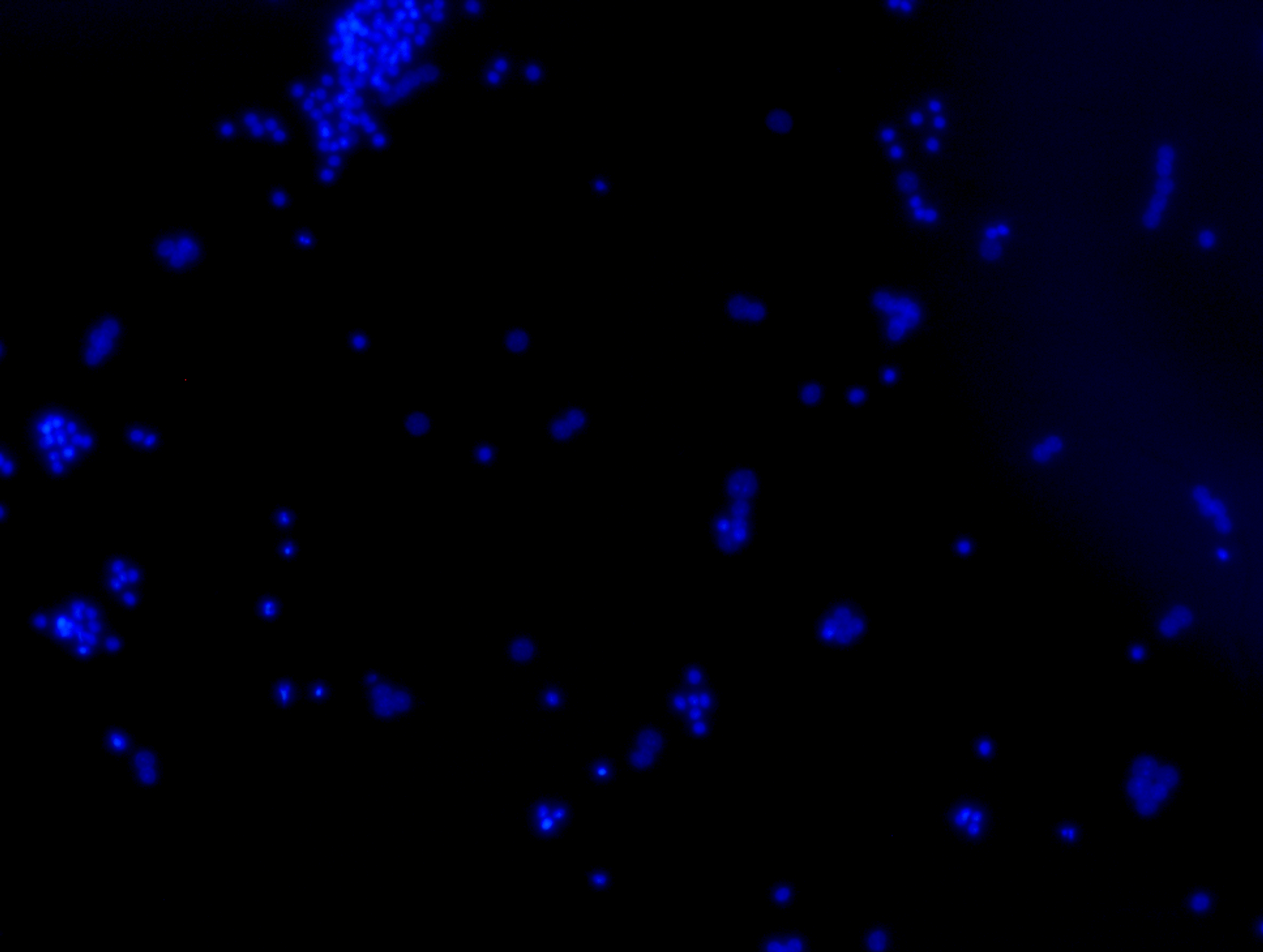

Supplement: Figure 3—source data 2. [file elife-92142-fig3-data2.zip › Source data 2-The raw microscopy images for Figure 3/Figure 3G/RAW RANKL (1).tif]

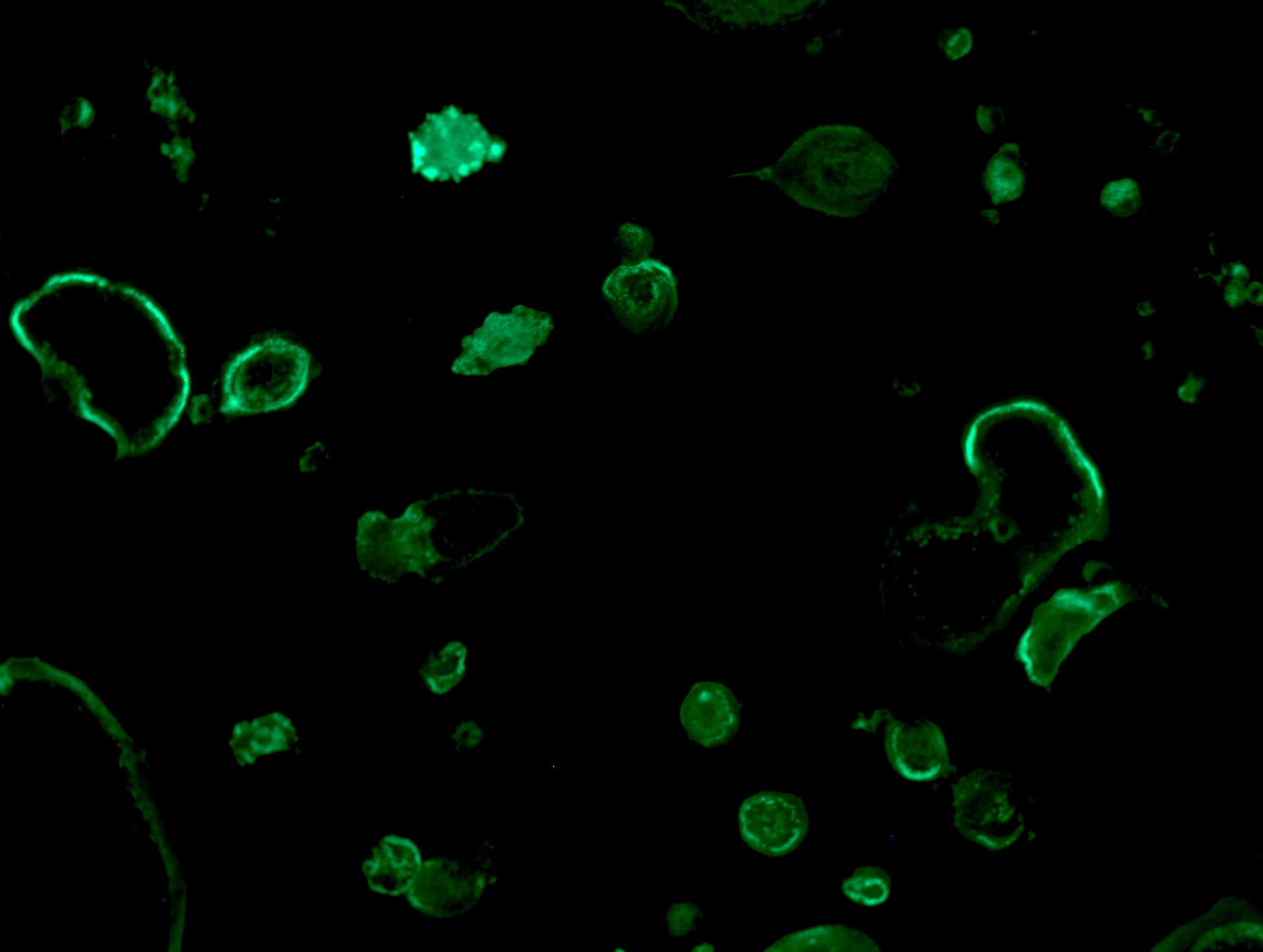

Supplement: Figure 3—source data 2. [file elife-92142-fig3-data2.zip › Source data 2-The raw microscopy images for Figure 3/Figure 3G/RAW GANT58 (3).tif]

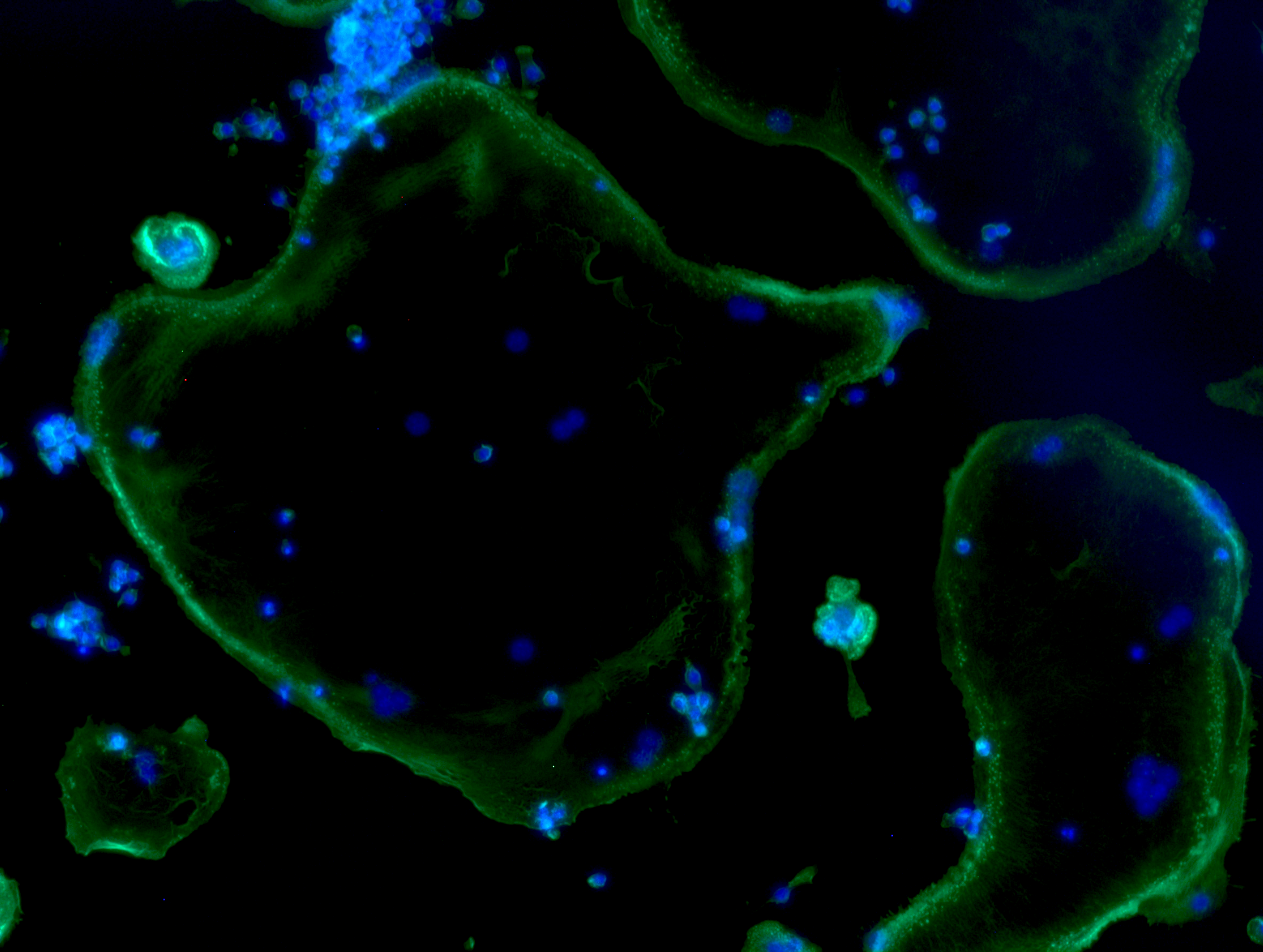

Supplement: Figure 3—source data 2. [file elife-92142-fig3-data2.zip › Source data 2-The raw microscopy images for Figure 3/Figure 3G/RAW RANKL (2).tif]

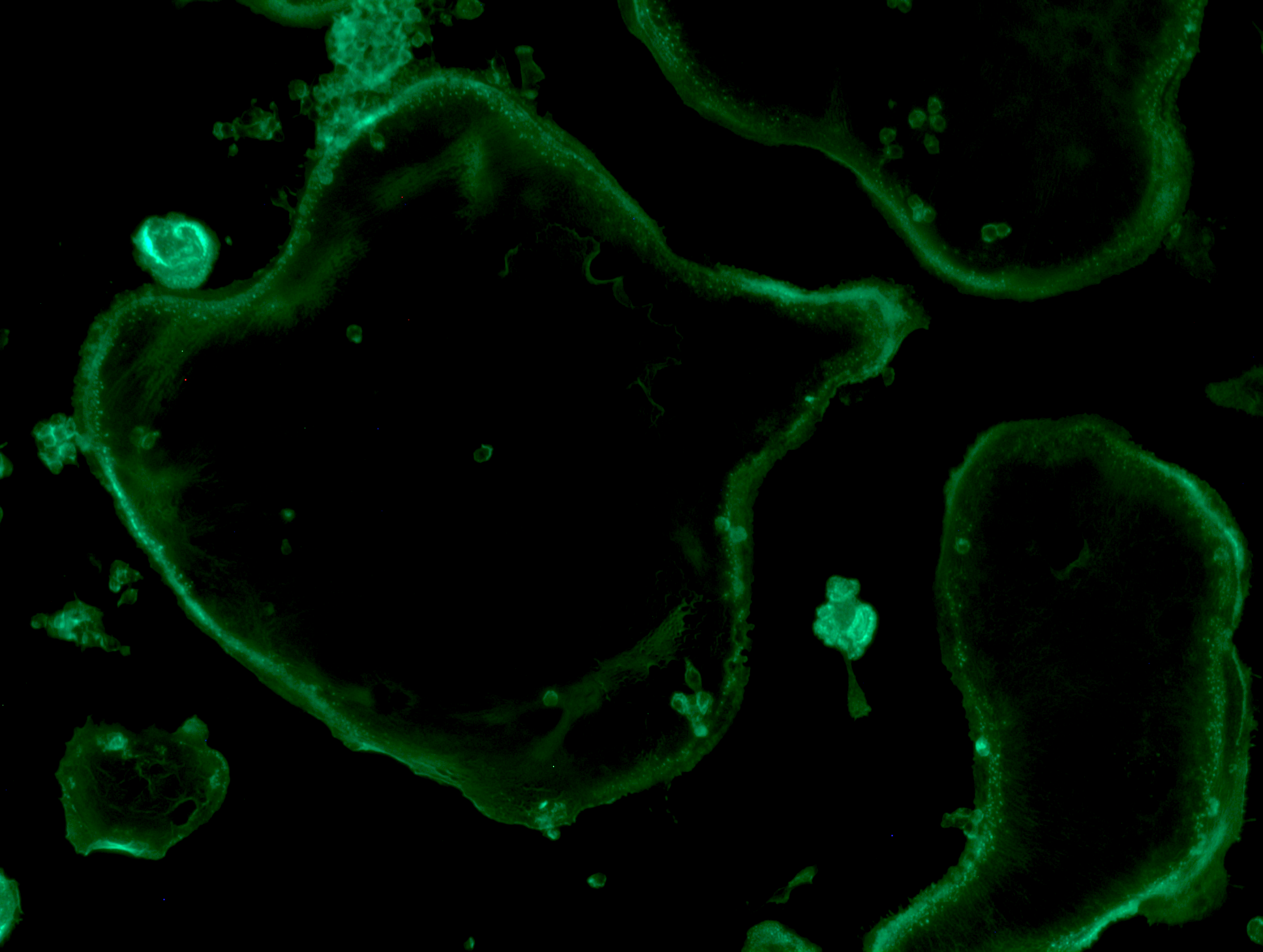

Supplement: Figure 3—source data 2. [file elife-92142-fig3-data2.zip › Source data 2-The raw microscopy images for Figure 3/Figure 3G/RAW RANKL (3).tif]

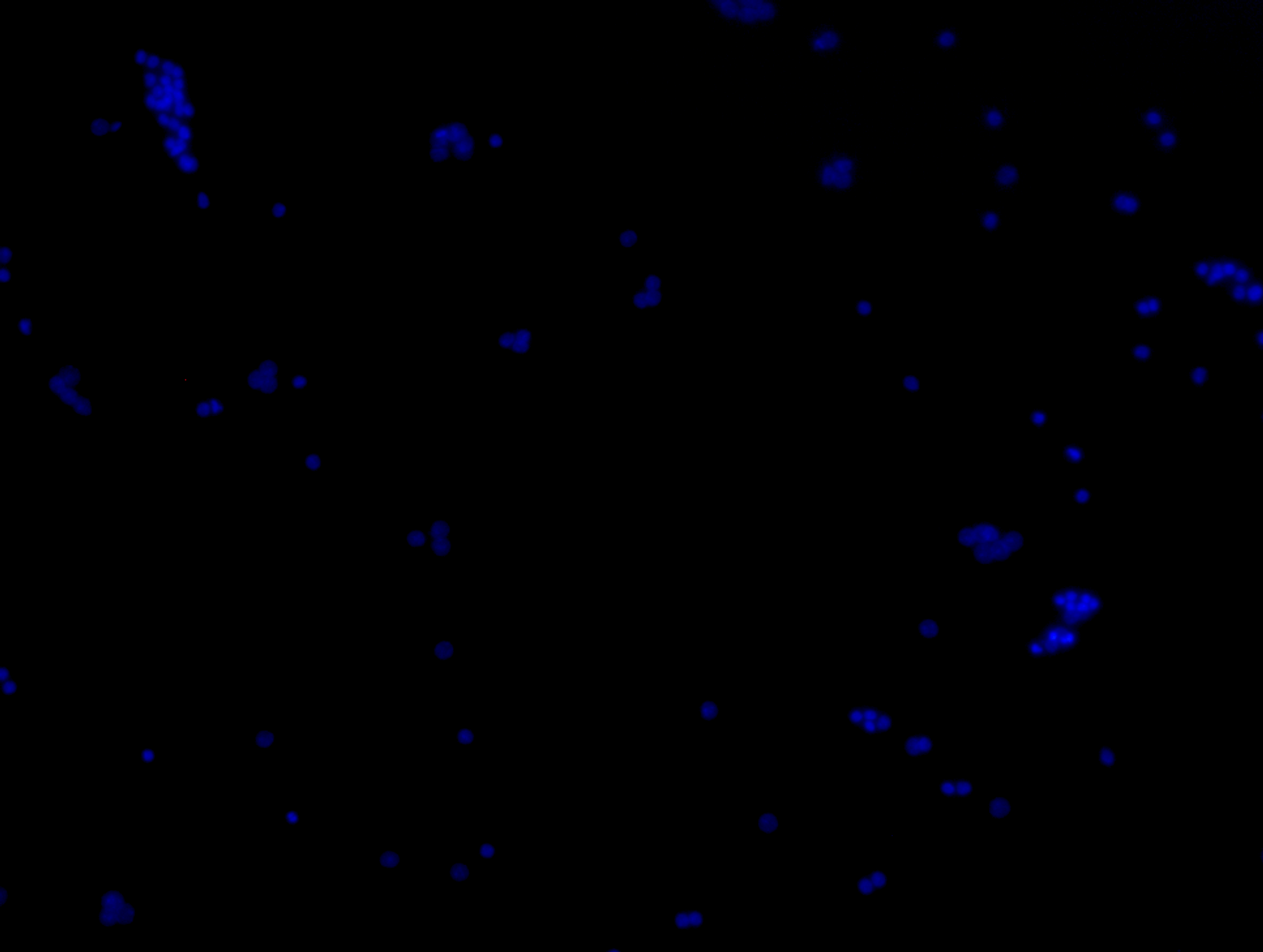

Supplement: Figure 3—source data 2. [file elife-92142-fig3-data2.zip › Source data 2-The raw microscopy images for Figure 3/Figure 3G/RAW GANT58 (1).tif]

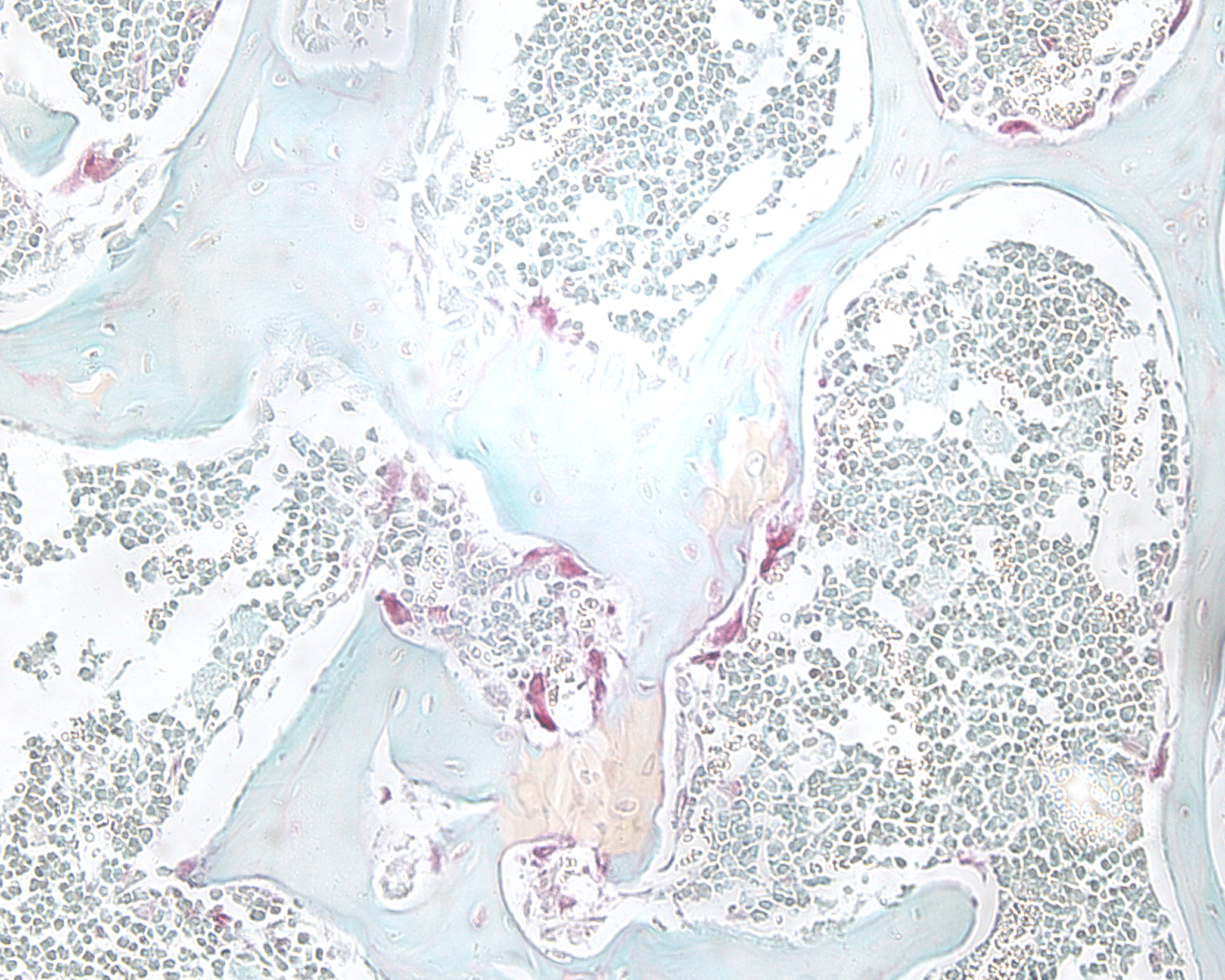

Supplement: Figure 3—source data 2. [file elife-92142-fig3-data2.zip › Source data 2-The raw microscopy images for Figure 3/Figure 3N/Vehicle/Vehicle-Zoom.tif]

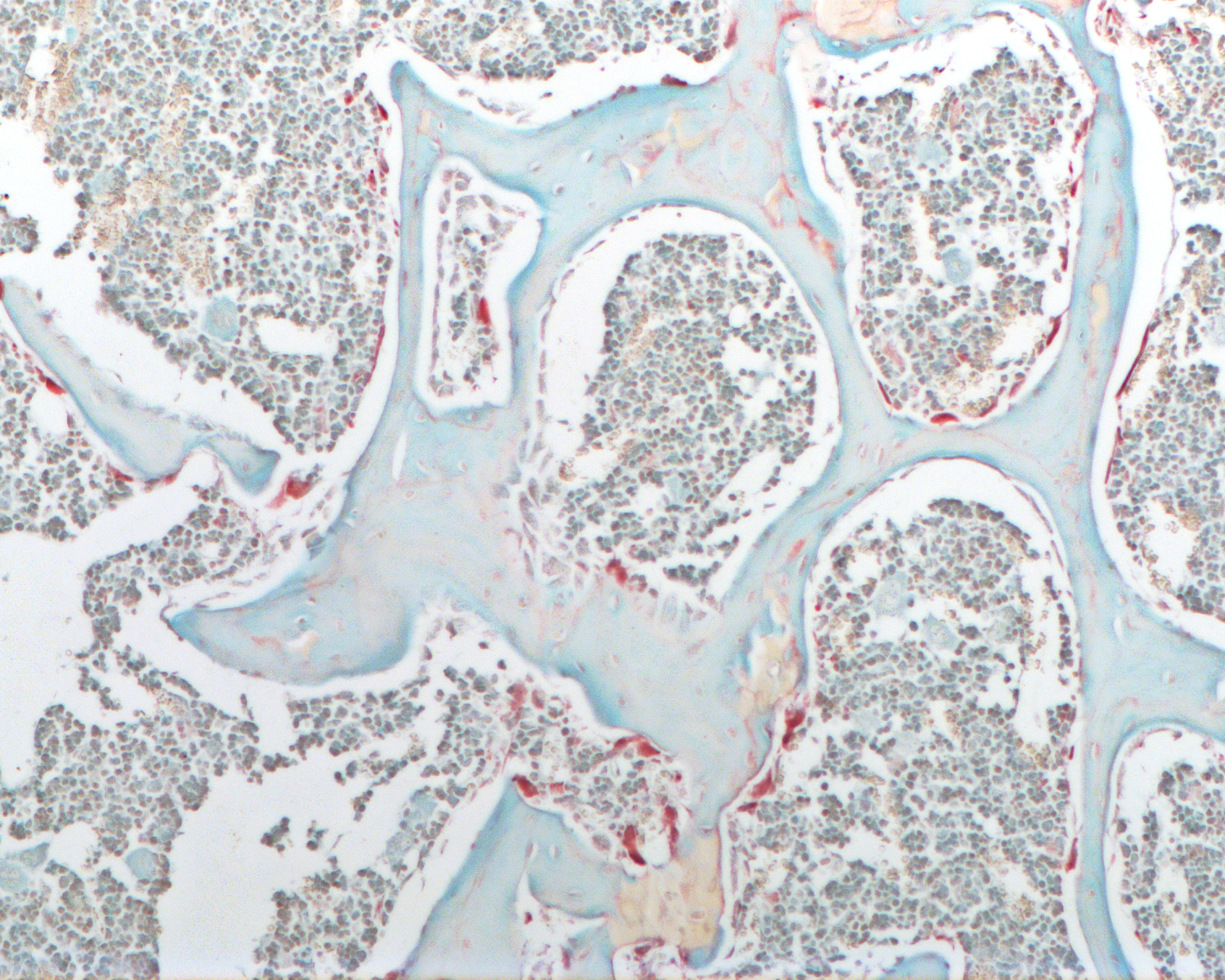

Supplement: Figure 3—source data 2. [file elife-92142-fig3-data2.zip › Source data 2-The raw microscopy images for Figure 3/Figure 3N/Vehicle/Vehicle.tif]

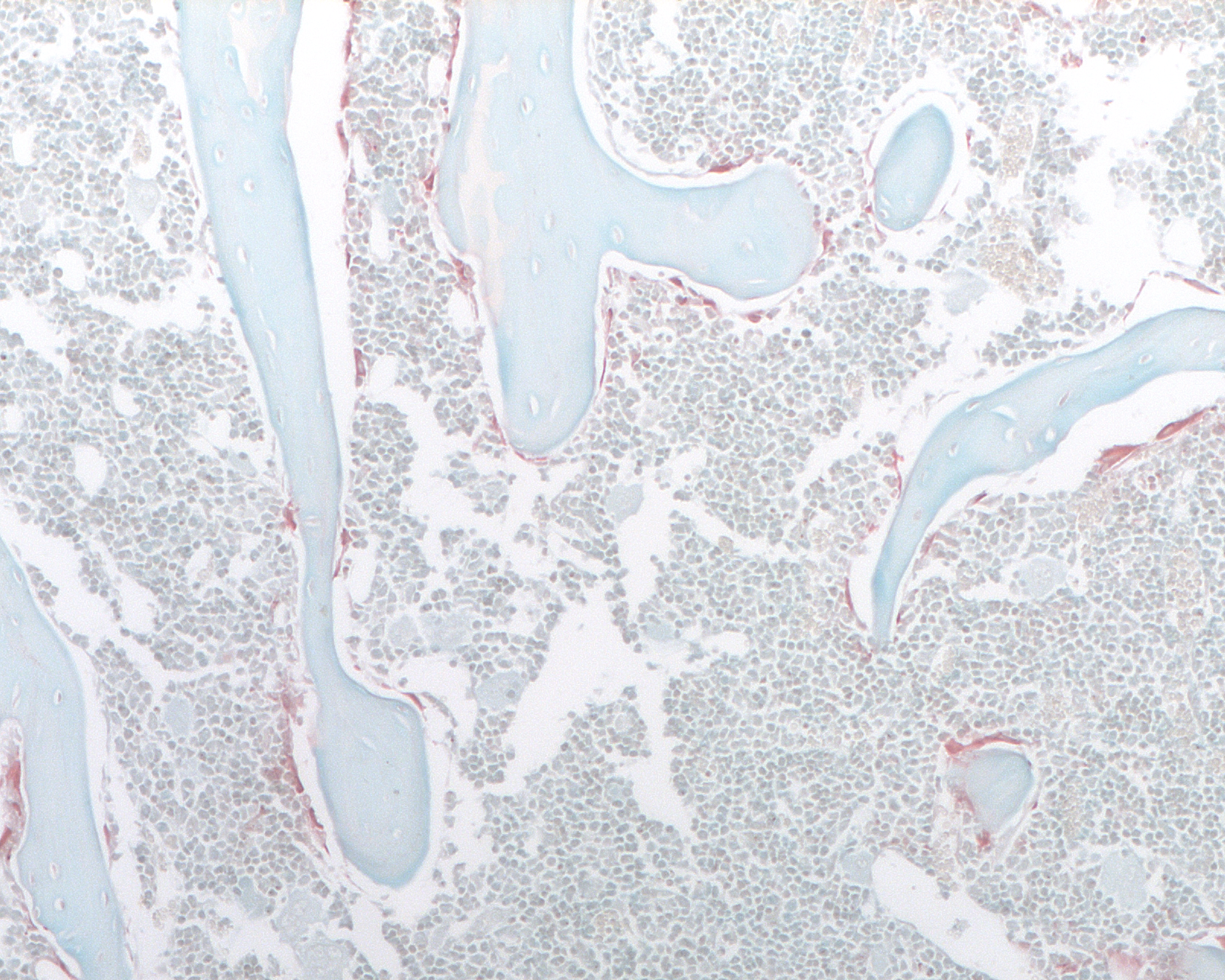

Supplement: Figure 3—source data 2. [file elife-92142-fig3-data2.zip › Source data 2-The raw microscopy images for Figure 3/Figure 3N/GANT58/GANT58.tif]

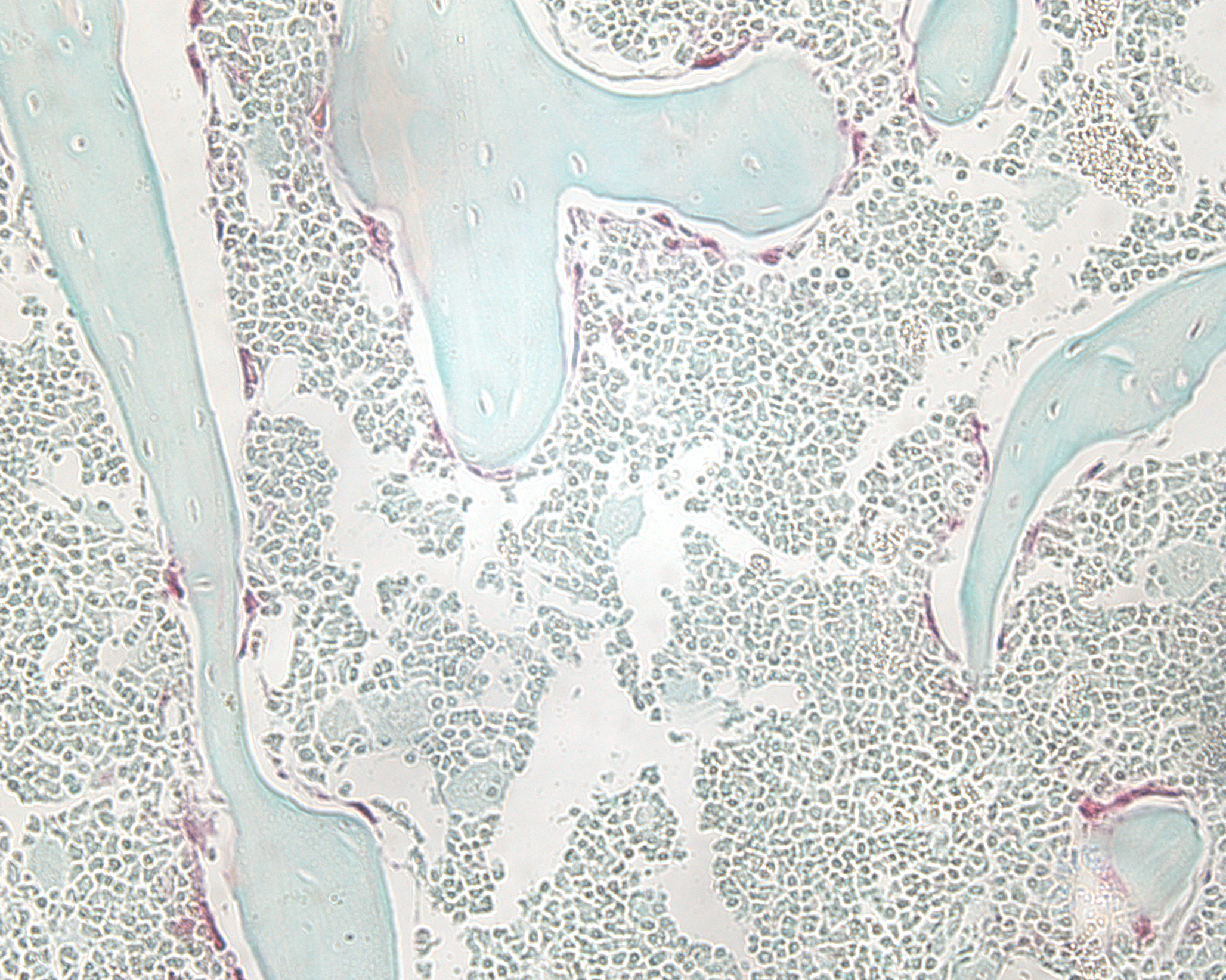

Supplement: Figure 3—source data 2. [file elife-92142-fig3-data2.zip › Source data 2-The raw microscopy images for Figure 3/Figure 3N/GANT58/GANT58-Zoom.tif]

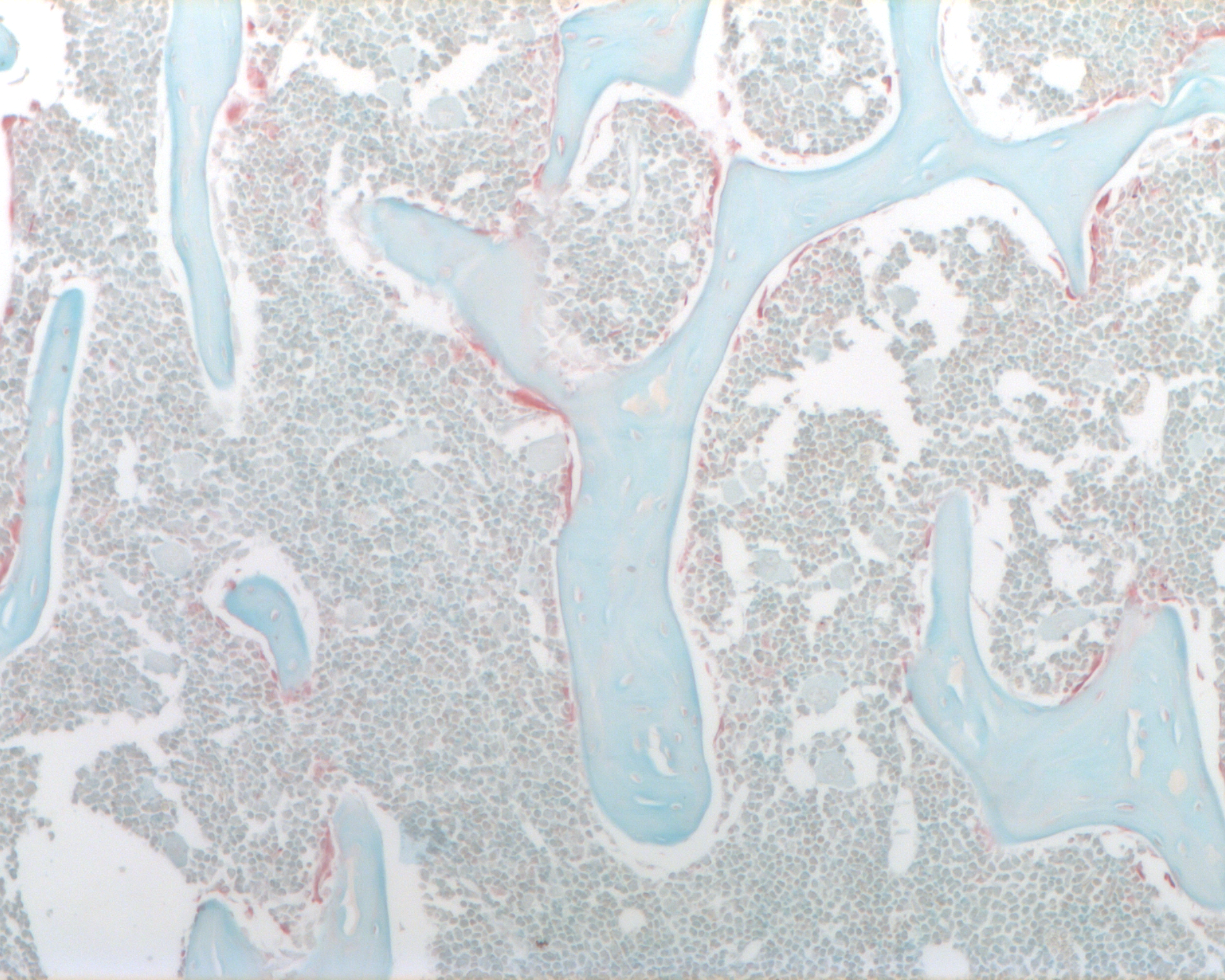

Supplement: Figure 3—source data 2. [file elife-92142-fig3-data2.zip › Source data 2-The raw microscopy images for Figure 3/Figure 3N/Sham/Sham.tif]

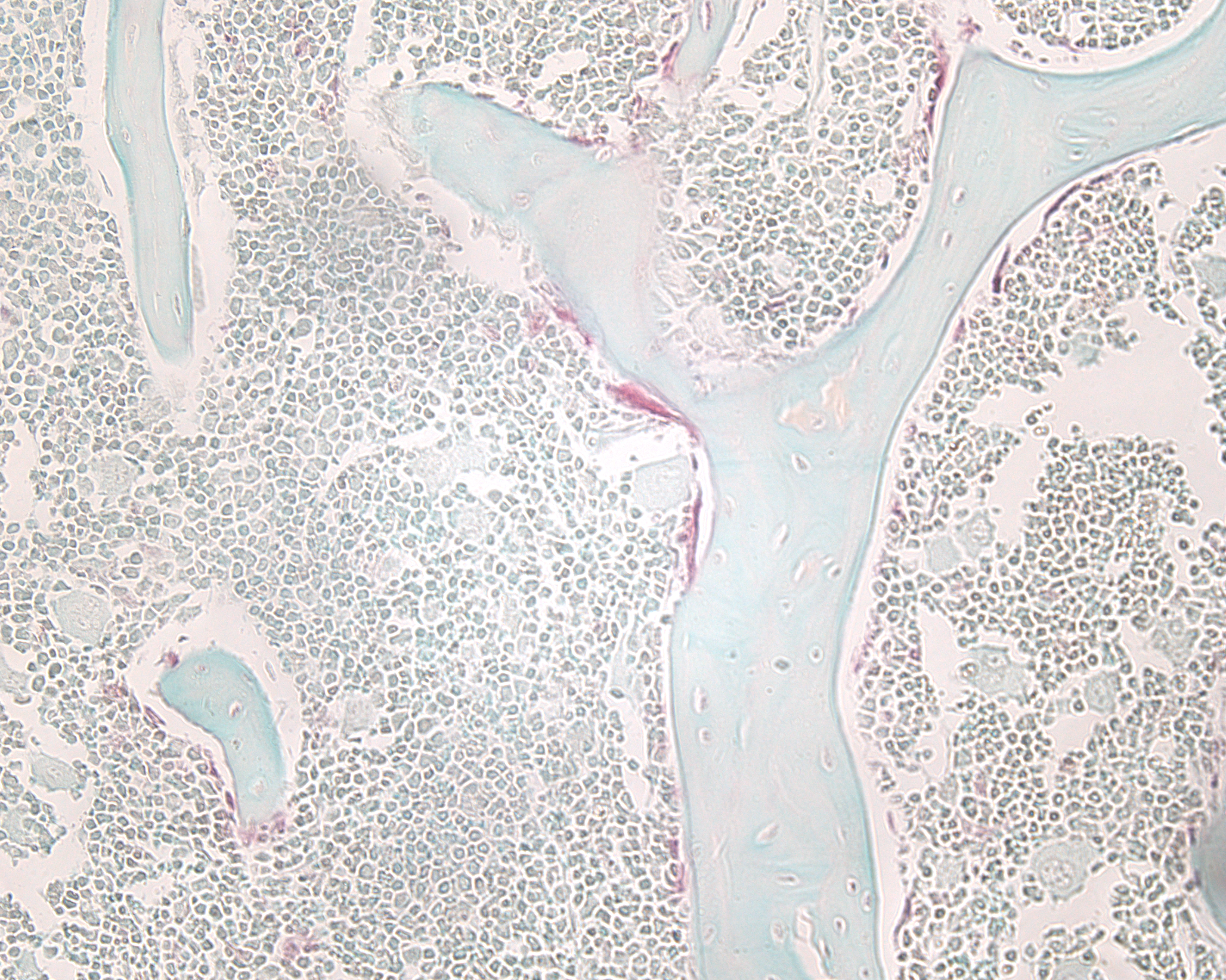

Supplement: Figure 3—source data 2. [file elife-92142-fig3-data2.zip › Source data 2-The raw microscopy images for Figure 3/Figure 3N/Sham/Sham-Zoom.tif]

## Slide 1
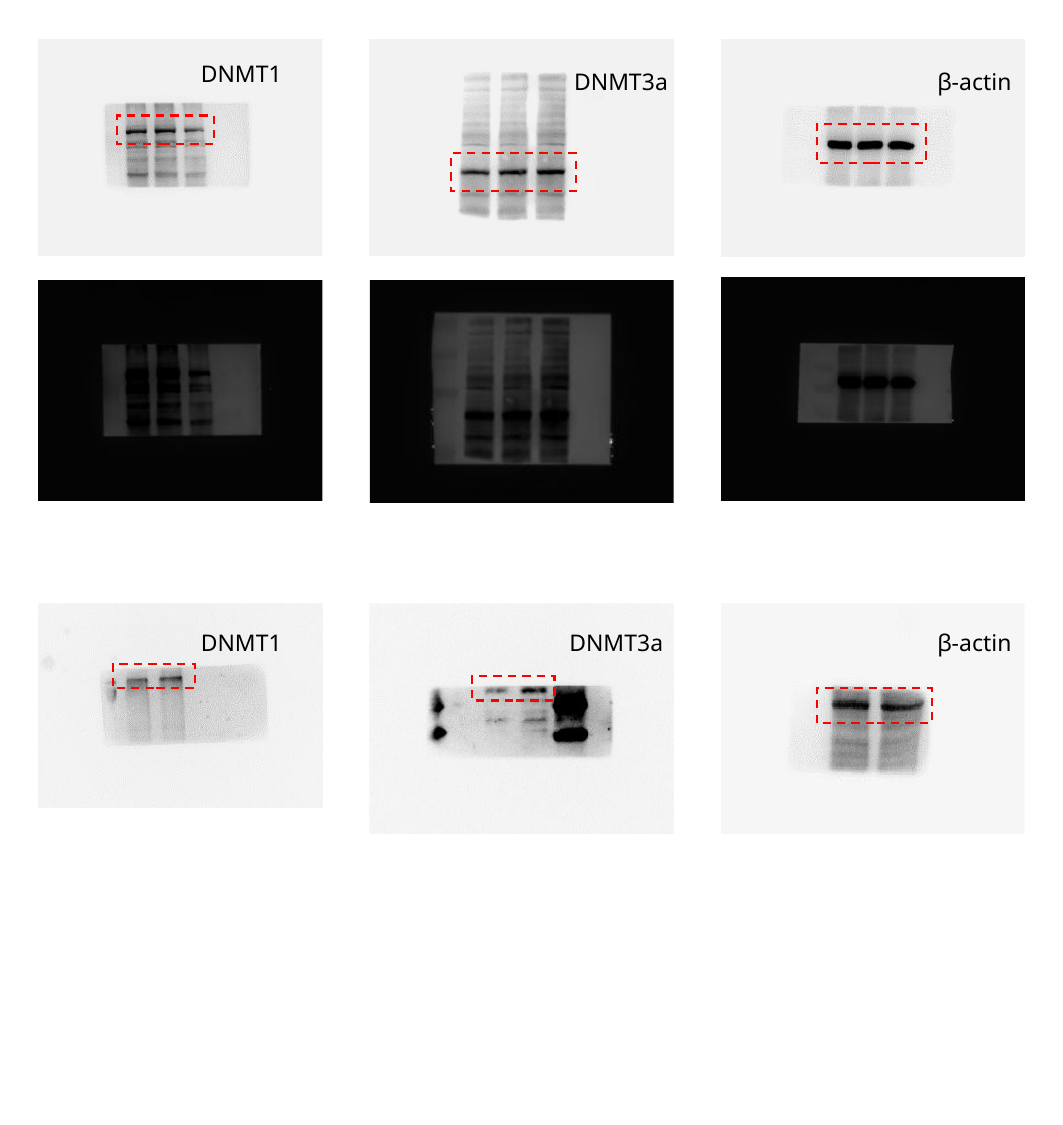

DNMT1
DNMT3a
β-actin
DNMT1
DNMT3a
β-actin

Supplement: Figure 5—source data 1. [file elife-92142-fig5-data1.pptx]

## Slide 1
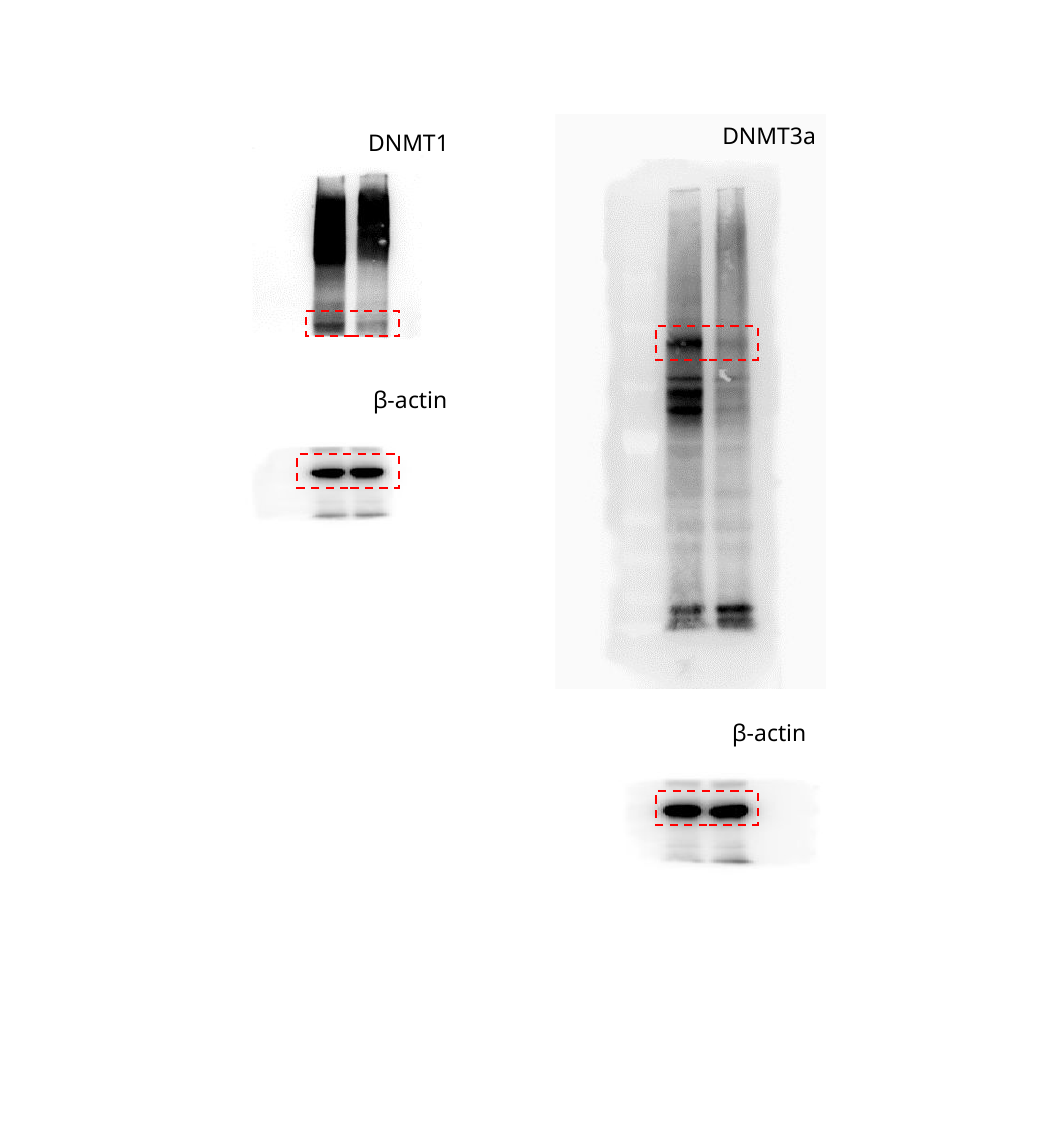

DNMT3a
DNMT1
β-actin
β-actin

Supplement: Figure 5—figure supplement 2—source data 1. [file elife-92142-fig5-figsupp2-data1.pptx]

## Slide 1
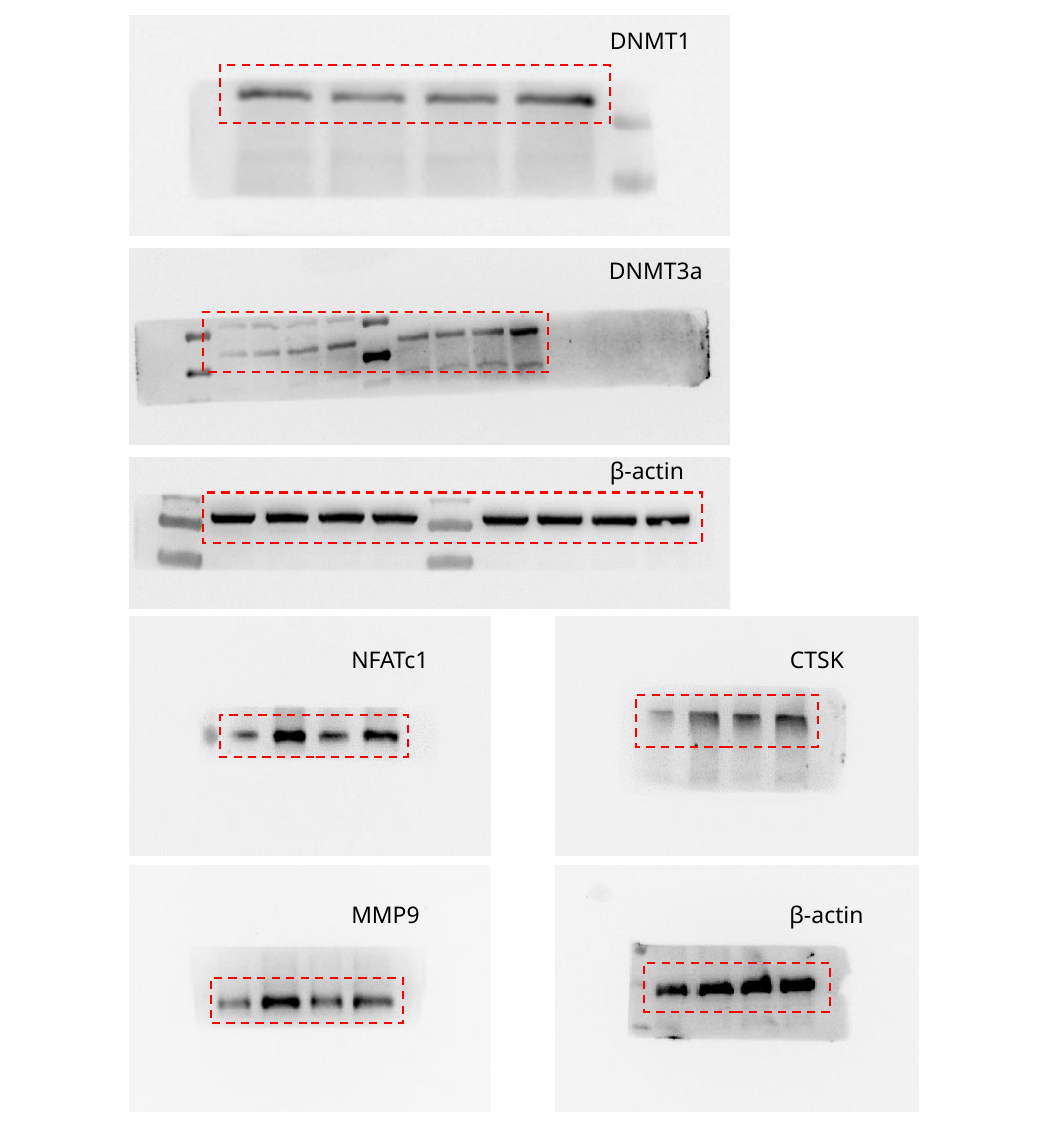

DNMT1
DNMT3a
β-actin
NFATc1
CTSK
MMP9
β-actin

Supplement: Figure 6—source data 1. [file elife-92142-fig6-data1.pptx]
