# Supplementary material for: GLI1 facilitates collagen-induced arthritis in mice by collaborative regulation of DNA methyltransferases
Source: eLife. 2023 Nov 6;12:e92142. doi: 10.7554/eLife.92142 (PMC10627516; doi:10.7554/eLife.92142)
Supplement: Figure 4—figure supplement 2—source data 1. [file elife-92142-fig4-figsupp2-data1.pptx]

## Slide 1
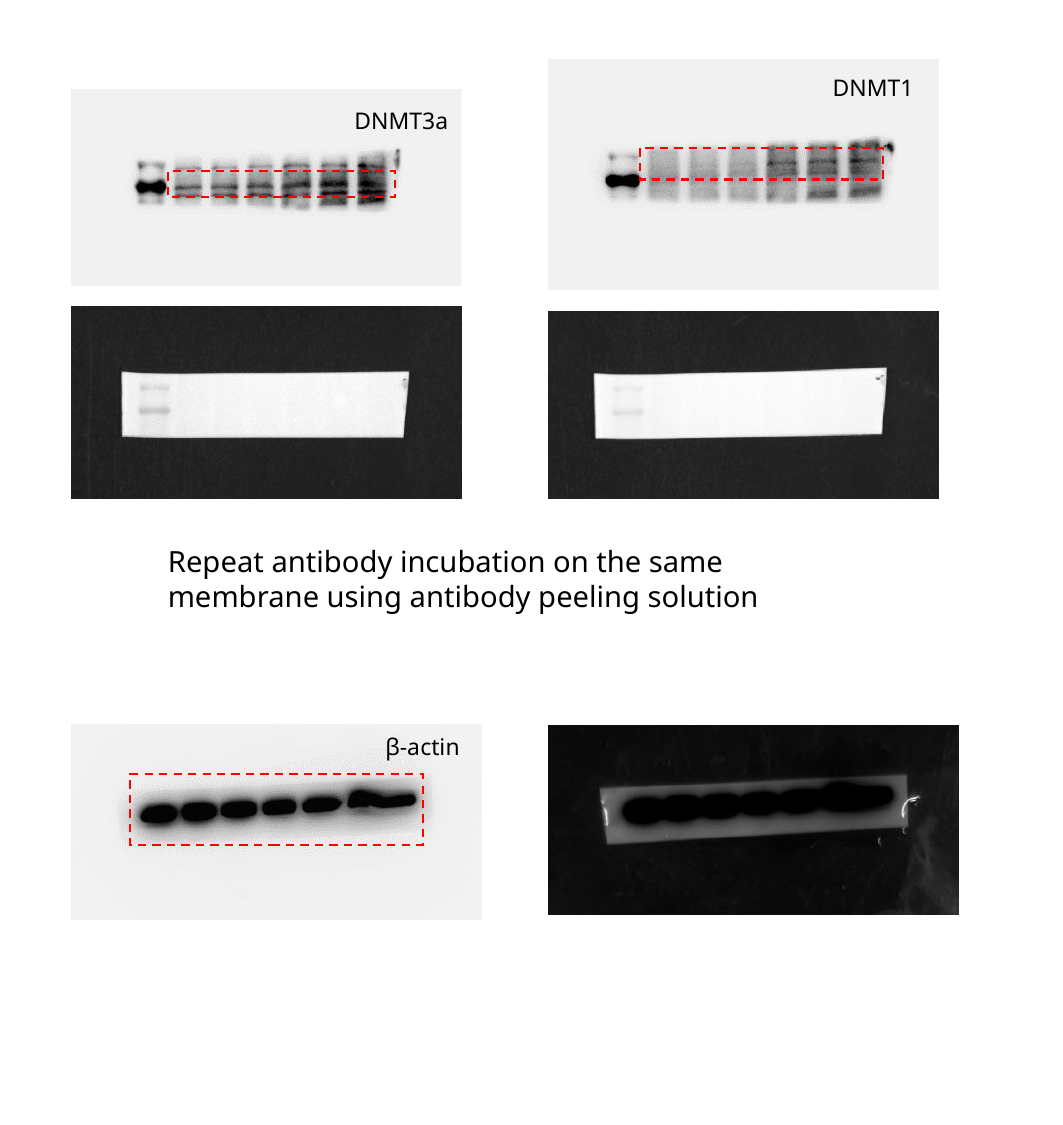

DNMT1
DNMT3a
Repeat antibody incubation on the same membrane using antibody peeling solution
β-actin
