# Supplementary material for: GLI1 facilitates collagen-induced arthritis in mice by collaborative regulation of DNA methyltransferases
Source: eLife. 2023 Nov 6;12:e92142. doi: 10.7554/eLife.92142 (PMC10627516; doi:10.7554/eLife.92142)
Supplement: Supplementary file 1. [file elife-92142-supp1.docx]

**Table supplement 1. siRNA target sequences.**

| **siRNA target sequences** | | |
| --- | --- | --- |
| **Gene** | **sense (5’-3’)** | **antisense (5’-3’)** |
| *Gli1-mus-#1* | GCCUUACAUGUGUGAGCAATT | UUGCUCACACAUGUAAGGCTT |
| *Gli1-Mus-#2* | CCUCGGAGUUCAGUCAAAUTT | AUUUGACUGAACUCCGAGGTT |
| *Gli1-mus-#3* | CCACCCUACCUCUGUCUAUTT | AUAGACAGAGGUAGGGUGGTT |
| *Dnmt3a-Mus* | CCAUGUACCGCAAAGCCAUTT | AUGGCUUUGCGGUACAUGGTT |
| *Dnmt1-Mus* | GUCUCAUUGAGAAGAAUGUTT | ACAUUCUUCUCAAUGAGACTT |
