# Supplementary material for: GLI1 facilitates collagen-induced arthritis in mice by collaborative regulation of DNA methyltransferases
Source: eLife. 2023 Nov 6;12:e92142. doi: 10.7554/eLife.92142 (PMC10627516; doi:10.7554/eLife.92142)
Supplement: Supplementary file 2. [file elife-92142-supp2.docx]

**Table supplement 2. Predictive analysis of protein binding.**

| **Interface Summary**      XML | | | | | | | | | |  |
| --- | --- | --- | --- | --- | --- | --- | --- | --- | --- | --- |
|  | | | | | | [Structure 1](javascript:openWindow('pi_ipage_struct1.html',400,250);) | | [Structure 2](javascript:openWindow('pi_ipage_struct2.html',400,250);) | |  |
| [Selection range](javascript:openWindow('pi_ipage_ssu.html',400,250);) | | | | | | A | | B | |  |
| **class** | | | | | | Protein | | Protein | |  |
| **symmetry operation** | | | | | | x,y,z | | ,, | |  |
| **symmetry ID** | | | | | | 1_555 | | 0_555 | |  |
| [Number of atoms](javascript:openWindow('pi_ipage_nat.html',400,250);) | | | | | |  |  |  |  |  |
| **interface** | | | | | | 107 | 1.00% | 57 | 82.60% |  |
| **surface** | | | | | | 5694 | 54.70% | 66 | 95.70% |  |
| **total** | | | | | | 10406 | 100.00% | 69 | 100.00% |  |
| [Number of residues](javascript:openWindow('pi_ipage_nres.html',400,250);) | | | | | |  |  |  |  |  |
| **interface** | | | | | | 33 | 2.50% | 10 | 100.00% |  |
| **surface** | | | | | | 1226 | 92.00% | 10 | 100.00% |  |
| **total** | | | | | | 1333 | 100.00% | 10 | 100.00% |  |
| [Solvent-accessible area, Å](javascript:openWindow('pi_ipage_surf.html',400,250);) | | | | | |  |  |  |  |  |
| **interface** | | | | | | 614 | 1.00% | 842.9 | 68.50% |  |
| **total** | | | | | | 59982.9 | 100.00% | 1229.6 | 100.00% |  |
| [Solvation energy, kcal/mol](javascript:openWindow('pi_ipage_se.html',400,250);) | | | | | |  |  |  |  |  |
| **isolated structure** | | | | | | -1184.8 | 100.00% | -4.8 | 100.00% |  |
| **gain on complex formation** | | | | | | -2 | 0.20% | -5.8 | 120.00% |  |
| **average gain** | | | | | | -2.6 | 0.20% | -4.7 | 97.60% |  |
| **P-value** | | | | | | 0.573 |  | 0.344 |  |  |
| **Hydrogen bonds**      XML | | | | | | | |  |  |  |
| **##** | | [Structure 1](javascript:openWindow('pi_ipage_atom1.html',400,250);) | [Dist. [Å]](javascript:openWindow('pi_ipage_atmdist.html',400,250);) | | [Structure 2](javascript:openWindow('pi_ipage_atom2.html',400,250);) | | |  |  |  |
| 1 | | A:SER 267[ N  ] | 2.89 | | B:SER 120[ O  ] | | |  |  |  |
| 2 | | A:SER 267[ OG ] | 3.23 | | B:SER 120[ O  ] | | |  |  |  |
| 3 | | A:GLY 268[ N  ] | 3.80 | | B:SER 120[ O  ] | | |  |  |  |
| 4 | | A:ASN 265[ ND2] | 3.02 | | B:SER 120[ OG ] | | |  |  |  |
| 5 | | A:HIS 160[ N  ] | 3.11 | | B:TYR 121[ O  ] | | |  |  |  |
| 6 | | A:HIS 160[ ND1] | 3.87 | | B:TYR 121[ O  ] | | |  |  |  |
| 7 | | A:SER 270[ N  ] | 2.83 | | B:GLY 122[ O  ] | | |  |  |  |
| 8 | | A:SER 162[ N  ] | 3.15 | | B:HIS 123[ O  ] | | |  |  |  |
| 9 | | A:SER 162[ OG ] | 3.72 | | B:SER 125[ OG ] | | |  |  |  |
| 10 | | A:ASN 265[ O  ] | 2.95 | | B:SER 120[ N  ] | | |  |  |  |
| 11 | | A:ASP 159[ OD2] | 2.99 | | B:TYR 121[ N  ] | | |  |  |  |
| 12 | | A:GLY 268[ O  ] | 3.04 | | B:GLY 122[ N  ] | | |  |  |  |
| 13 | | A:HIS 160[ O  ] | 2.89 | | B:HIS 123[ N  ] | | |  |  |  |
| 14 | | A:TYR 147[ OH ] | 2.72 | | B:HIS 123[ ND1] | | |  |  |  |
| 15 | | A:ASP 159[ OD1] | 2.94 | | B:HIS 123[ NE2] | | |  |  |  |
| 16 | | A:SER 270[ O  ] | 3.08 | | B:LEU 124[ N  ] | | |  |  |  |
| 17 | | A:SER 162[ OG ] | 2.81 | | B:SER 125[ N  ] | | |  |  |  |
| 18 | | A:GLU 376[ OE2] | 2.61 | | B:SER 125[ OG ] | | |  |  |  |
| 19 | | A:SER 162[ O  ] | 3.82 | | B:ILE 126[ N  ] | | |  |  |  |
| **Salt bridges**      XML | | | | | | | |  |  |  |
| **##** | [Structure 1](javascript:openWindow('pi_ipage_atom1.html',400,250);) | | [Dist. [Å]](javascript:openWindow('pi_ipage_atmdist.html',400,250);) | [Structure 2](javascript:openWindow('pi_ipage_atom2.html',400,250);) | | | |  |  |  |
| 1 | A:ASP 159[ OD1] | | 2.94 | B:HIS 123[ NE2] | | | |  |  |  |
|  | | | | | | | |  |  |  |
|  | | | | | | | |  |  |  |
