# Supplementary material for: GLI1 facilitates collagen-induced arthritis in mice by collaborative regulation of DNA methyltransferases
Source: eLife. 2023 Nov 6;12:e92142. doi: 10.7554/eLife.92142 (PMC10627516; doi:10.7554/eLife.92142)
Supplement: Supplementary file 3. [file elife-92142-supp3.docx]

**Table supplement 3.** **RT-qPCR primer sequence.**

| **Primer sequence** | | |
| --- | --- | --- |
| **mRNA** | **Forward (5’-3’)** | **Reverse (5’-3’)** |
| *Il6* | CCCGGAGGAGACTTCAG | CAGATTGCCATTGCACAAC |
| *Il1b* | ACTCATTGTGGCTGTGGAGA | TTGTTCATCTCGGAGCCTGT |
| *Tnfa* | CCAAAGGGATGAGAAGTTCC | CTCCACTTGGTGGTTTGCTA |
| *Il10* | GAGAAGCATGGCCCAGAAATC | CGCATCCTGAGGGTCTTCA |
| *Gli1* | GGTGTGTAATTACGTTCAGTCG | GGATAGGAGCCTGATTTGTGAT |
| *Dnmt1* | GAGACGAAAAACGACACGTAAA | CACTTTGGTGAGTTGATCTTCG |
| *Dnmt3a* | GATGATCGAAAGGAAGGAGAGG | TTCTCCAAGTCTCCATTGGGTA |
| *Actb* | CTACCTCATGAAGATCCTGACC | CACAGCTTCTCTTTGATGTCAC |
